# Pain medication tapering for patients with Persistent Spinal Pain Syndrome Type II, treated with Spinal Cord Stimulation: A RCT–study protocol of the PIANISSIMO study

**DOI:** 10.1371/journal.pone.0302842

**Published:** 2024-08-12

**Authors:** Maarten Moens, Cleo Lina Crunelle, Koen Putman, Elke Wuyts, Frenn Bultinck, Hubert Van Puyenbroeck, PIANISSIMO consortium, Lisa Goudman

**Affiliations:** 1 STIMULUS research group, Vrije Universiteit Brussel, Brussels, Belgium; 2 Department of Neurosurgery, Universitair Ziekenhuis Brussel, Brussels, Belgium; 3 Department of Radiology, Universitair Ziekenhuis Brussel, Brussels, Belgium; 4 Center for Neurosciences (C4N), Vrije Universiteit Brussel, Brussels, Belgium; 5 Pain in Motion Research Group (PAIN), Department of Physiotherapy, Human Physiology and Anatomy, Faculty of Physical Education & Physiotherapy, Vrije Universiteit Brussel, Brussels, Belgium; 6 Department of Psychiatry, Vrije Universiteit Brussel, University Hospital Brussels (UZ Brussel), Brussel, Belgium; 7 Faculty of Medicine and Pharmacy, Department of Public Health (GEWE), Interuniversity Centre for Health Economics Research (I-CHER), Vrije Universiteit Brussel, Brussels, Belgium; 8 Research Foundation Flanders (FWO), Brussel, Belgium; PLOS: Public Library of Science, UNITED KINGDOM

## Abstract

**Background:**

Spinal Cord Stimulation (SCS) may provide pain relief in patients with therapy-refractory Persistent Spinal Pain Syndrome Type II (PSPS-T2). Despite the evidence that SCS can reduce disability and reduce pain medication usage, only 25% of the patients is able to completely omit pain medication usage after 12 months of SCS. To tackle the high burden of patients who consume a lot of pain medication, tapering programs could be initiated before starting a trajectory with SCS. The current objective is to examine whether a pain medication tapering program before SCS alters disability in PSPS-T2 patients compared to no tapering program.

**Methods and design:**

A three-arm, parallel-group multicenter randomized controlled trial will be conducted including 195 patients who will be randomized (1:1:1) to either (a) a standardized pain medication tapering program, (b) a personalized pain medication tapering program, or (c) no tapering program before SCS implantation, all with a follow-up period until 12 months after implantation. The primary outcome is disability. The secondary outcomes are pain intensity, health-related quality of life, participation, domains affected by substance use, anxiety and depression, medication usage, psychological constructs, sleep, symptoms of central sensitization, and healthcare expenditure.

**Discussion:**

Within the PIANISSIMO project we propose a way to reduce the risks of adverse events, medication-induced hyperalgesia, tolerance, and dependence by providing pain medication tapering before SCS. Due to the lack of a commonly accepted in-hospital tapering approach, two different tapering programs will be evaluated in this study. If pain medication tapering programs are deemed to be more effective than no tapering on disability, this would add to the evidence towards an improved patient-centered care model in this patient group and set a clear path to advocate for pain medication tapering before SCS as the new standard treatment guideline for these patients.

**Trial registration:**

ClinicalTrials.gov NCT05861609. Registered on May 17, 2023.

## Introduction

When surgical spine interventions fail to provide sufficient pain relief, patients may remain with persistent pain, termed Persistent Spinal Pain Syndrome Type II (PSPS-T2). The pooled prevalence of patients experiencing chronic pain after spinal surgery is 14.9% (95% CI from 12.38 to 17.76) [1]. Patients with PSPS-T2 usually have a long-standing history of pain and are often prescribed opioids that might be dosed over five times the morphine equivalent daily dose [2] Despite recommendations and guidelines to avoid doses above 90 MME [morphine milligram equivalents) [3–5], more than 24.6% [6] up to 39.3% [7] of chronic non-cancer pain patients receives doses ≥ 90 MME. Long-term opioid usage is associated with constipation, tolerance, hyperalgesia, respiratory depression, sedation, increased risk for addiction and the prominent and aversive withdrawal symptoms that manifest when opioid usage is discontinued [8]. Avoidance of the opioid withdrawal syndrome is a frequently cited reason for continued opioid usage [9]. Besides the risks of opioids, adverse drug reaction reports of misuse, abuse, and dependence of gabapentinoids were revealed in a time series from 2006 to 2015 [10]. Moreover, the misuse of gabapentin produces anxiolytic effects, with risks of physiologic dependence and withdrawal syndrome [11]. Additionally, a recent study revealed that in 2022, the prevalence of prescription of neuropathic mood agents was 78.5% and 50.6% for benzodiazepines in this population [12]. Thus, a complex clinical challenge has emerged since reducing pain medication is likely to increase patient’s pain intensity, whereas increasing or sustaining doses will lead to changes in pain sensitivity and increases the risk of substance use dependence [13].

When conservative treatment does not achieve adequate pain relief, Spinal Cord Stimulation (SCS) is proposed as a minimally invasive treatment option [14]. The effectiveness of SCS has already been proven for reducing low back and leg pain, increasing functionality, and optimizing health-related quality of life [15–18]. Additionally, SCS is cost-effective for patients with PSPS-T2 [19]. Recently, a meta-analysis evaluated the effect of SCS on opioid intake and overall pain medication reduction in patients with intractable low back and/or leg pain [20]. The authors reported that when being treated with SCS, the odds of reducing opioid consumption were significantly increased compared to conventional medical care (OR 8.60 (95% CI from 1.93 to 38.30)) [20]. Nevertheless, despite a significant decrease in the amount of opioids, the number of patients that could effectively reduce or fully eliminate opioid usage after SCS, was rather limited (ranging from only 7 to 41%) [20]. Similarly, a multicenter cohort study in Belgium compared pain medication usage before SCS and after 12 months of SCS, which demonstrated a statistically significant decrease in pain medication usage [21]. However, the actual number of patients taking no pain medication was still rather low, i.e., 9.3% at baseline versus 24.7% after 12 months of SCS [21].

In patients with intrathecal opioid therapy, Grider et al. revealed the efficacy of a pretrial elimination of systemic opioids followed by a period of abstinence [22]. In 2018, a retrospective study in 60 patients with chronic non-cancer pain confirmed the success rate of this concept [23]. When evaluating patients 1 year after SCS implantation, compared to pre-implantation, patients who reduced/eliminated opioids showed significantly improved functionality (decreased disability). In patients who remained on their pre-implantation opioid dosage, no differences in disability were observed [24]. In line with this reasoning, it is expected that the results of SCS will be further improved when patients are no longer taking pain medication before SCS implantation. This study aims to eliminate all usage of opioids and gabapentinoids and improve the probability of remaining opioid free and gabapentinoids free 12 months after implantation and as such obtaining a higher functionality.

### Objectives

The primary objective of the study is to examine whether there is a difference in disability after 12 months of SCS in patients with PSPS-T2 after receiving a standardized pain medication tapering protocol before SCS implantation, a personalized pain medication tapering protocol before SCS implantation, or no pain medication tapering protocol before SCS implantation. The secondary objective of the study is to examine whether there is a difference after 12 months of SCS in patients with PSPS-T2 after receiving a standardized pain medication tapering protocol before SCS implantation, a personalized pain medication tapering protocol before SCS implantation, or no tapering protocol before SCS implantation in terms of pain intensity, health-related quality of life, participation, domains affected by substance use, anxiety and depression, medication usage, psychological constructs, sleep, symptoms of central sensitization, and healthcare expenditure.

## Materials and methods

### Trial design

**Fig 1:**
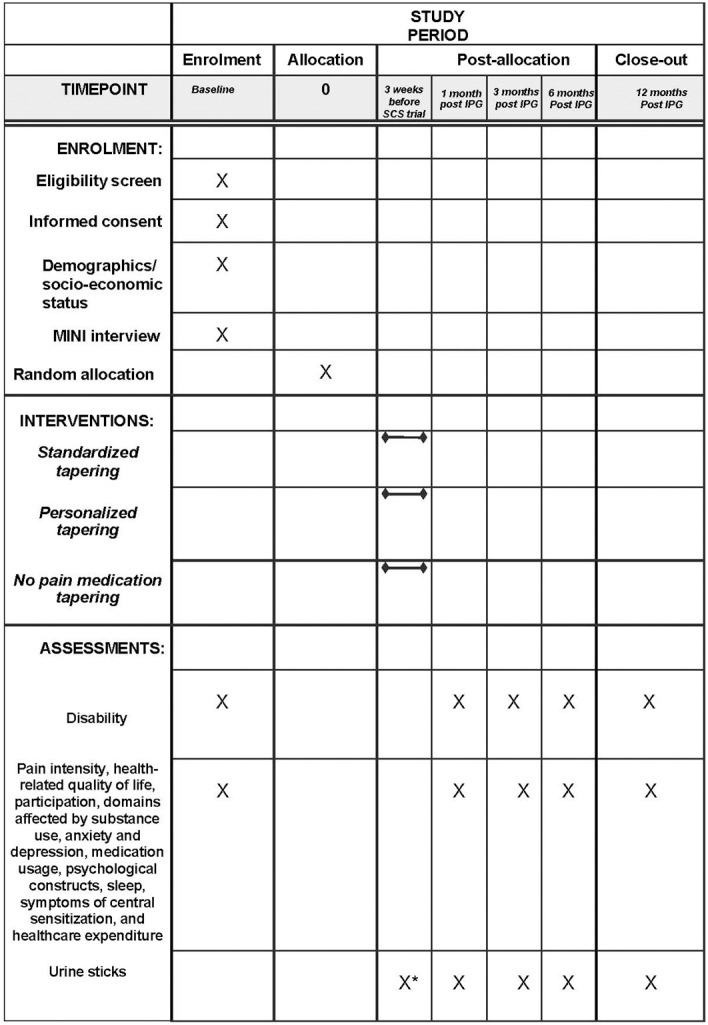
Participant timeline (SPIRIT). *: Evaluated at admission for SCS trial implantation. Abbreviations. IPG: Implantable pulse generator, MINI: Mini International Neuropsychiatric Interview; SCS: Spinal Cord Stimulation.

PIANISSIMO is a three-arm, parallel-group multicenter randomized controlled trial to evaluate whether pain medication tapering before SCS implantation compared to no pain medication tapering alters disability after 12 months in PSPS-T2 patients scheduled for SCS implantation (difference design). Patients will be randomized (1:1:1) to a) a standardized pain medication tapering program, b) a personalized pain medication program with temporary substitution therapy or c) no pain medication tapering program, all three with a follow-up period of 12 months after implantable pulse generator (IPG) implantation. The participant timeline for PIANISSIMO is presented in Fig 1.

### Study setting

The study will be conducted in three hospitals (Universitair Ziekenhuis Brussel (academic hospital), AZ Delta (regional hospital) and Heilig Hart Ziekenhuis Lier (regional hospital)) in Belgium. Additional sites will be contacted to participate in this study as well. All study sites are located in Belgium. Details on study sites can be found at ClinicalTrials.gov with Identifier: NCT05861609, May 17^th^, 2023. The study protocol was approved by the central Ethics Committee of the Universitair Ziekenhuis Brussel / Vrije Universiteit Brussel (EUDRACT: 2022-003925-23) and the Ethics Committees of each participating center. This study is funded by Research Foundation Flanders (FWO), Belgium (project number T000222N).

### Eligibility criteria

Patients being diagnosed with PSPS-T2, defined as patients suffering from neuropathic pain of radicular origin with pain in the lower back and/or leg(s), of an intensity of at least 4/10 on the Numeric Rating Scale, for a period of at least 6 months after a minimum of one anatomically successful spinal surgery and being refractory to conservative treatment (according to Belgian reimbursement rules from January 1st, 2018) will be eligible to participate. The inclusion criteria are:

- being diagnosed with PSPS-T2 and scheduled for SCS,
- being at least 18 years old,
- taking opioids,
- being able to speak and read Dutch or French.

Exclusion criteria include the following:

- being actively treated for cancer,
- having a life expectancy below 6 months,
- receiving intrathecal drug delivery,
- having contraindications for Clonidine (e.g., known hypotension which requires medication) or for Buprenorphine/Naloxone (e.g., severe respiratory insufficiency, hepatic insufficiency)
- having epilepsy currently treated by Pregabalin,
- currently using benzodiazepines at doses more than 40 mg diazepam-equivalents per day.

### Who will take informed consent?

Patients will be informed by their treating neurosurgeon or anesthesiologist about the study. If the patient is potentially eligible for the study and interested, the physician will provide the patient’s contact details to the study team. An investigator will then contact the patient by telephone to further inform eligible patients about the project and to screen the patient for in- and exclusion criteria. Patients who are then eligible for participation (based on the telephone interview) and are willing to participate, will receive detailed oral and written information about the study and have the opportunity to ask questions. Subsequently, patients will be asked to provide written informed consent before participation.

### Additional consent provisions for collection and use of participant data and biological specimens

A process evaluation will be conducted to supplement the primary trial results (by providing contextual information to the trial results and increasing the acceptability of the trial) and analyze secondary factors that made the tapering programs (un)successful [25]. The process evaluation is nested within the PIANISSIMO RCT and conducted according to process evaluation frameworks [26,27]. In parallel to the results of this RCT, a separate reporting will be created with the steps and results of this process evaluation. We will submit separate submissions to the Ethics Committee to obtain approval for the qualitative research that is incorporated in the process evaluation.

With respect to biological material, urinary samples will be collected, analyzed, and destroyed during the study visit.

### Interventions

**Fig 2:**
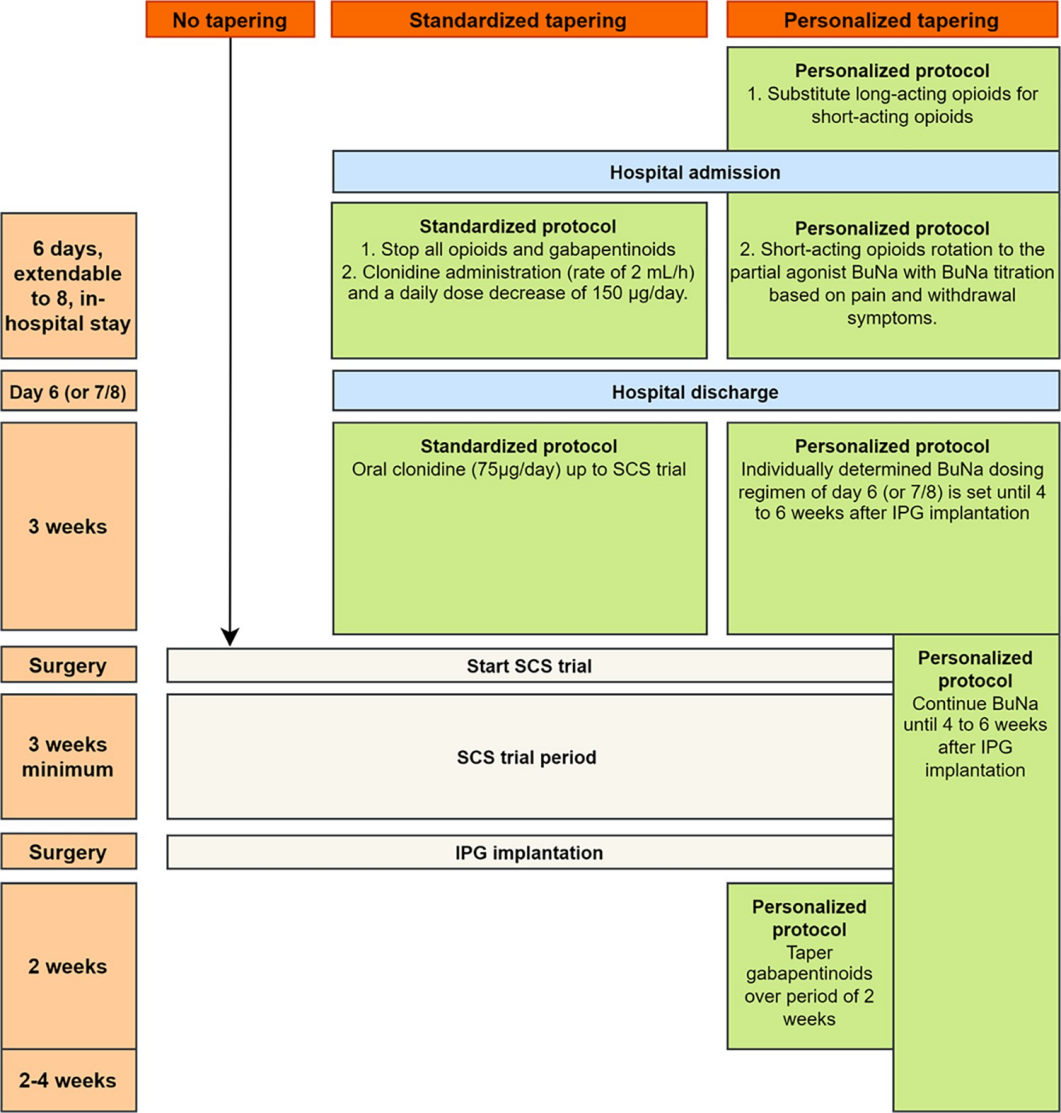
Overview of the treatment arms. Abbreviations. BuNA: Buprenorphine naloxone; IPG: Implantable pulse generator; SCS: Spinal Cord Stimulation.

Standardized pain medication tapering protocols are already implemented in Belgium in several centers, whereby the effectiveness, safety, and feasibility of this approach is already demonstrated, with tolerable withdrawal symptoms for patients [28]. In this standardized tapering program, Clonidine is administered to minimize the symptoms of withdrawal with a fixed dose per day [29,30]. In parallel, a different tapering protocol has been used in chronic non-cancer pain patients, in which opioid substitution treatment is implemented with the partial μ-opioid receptor agonist buprenorphine (analgesic ability), combined with naloxone [31,32]. Based on patient’s pain scores and severity of withdrawal symptoms, doses are individually regulated, leading to a personalized tapering protocol, with positive results on pain relief, opioid usage, opioid craving, and health-related quality of life [31,32]. For both tapering programs, patients will be hospitalized for six days. This might be extended to eight days, if deemed necessary according to the treating physician. The hospital stay is scheduled three weeks prior to the SCS trial. Both tapering programs will be compared to the usual care where no pain medication tapering is provided before SCS implantation (Fig 2).

### Control intervention: No pain medication tapering

When randomized to the control group, patients (N = 65) will not undergo a pain medication tapering program before SCS implantation. Patients first undergo a SCS trial period, followed by IPG implantation in case of a successful trial period (defined as 50% pain reduction and 50% reduction in pain medication usage, according to the current Belgium reimbursement rules). After implantation, patients are seen by the treating physician, pain nurse(s) and/or delegates of the companies of the SCS devices to program the SCS parameters. On top of that, they have a fixed follow-up appointment to re-evaluate the therapy every 6 months. Each hospital can continue usual care as normally provided to patients after SCS implantation.

### Experimental intervention: Standardized pain medication tapering protocol

**Fig 3:**
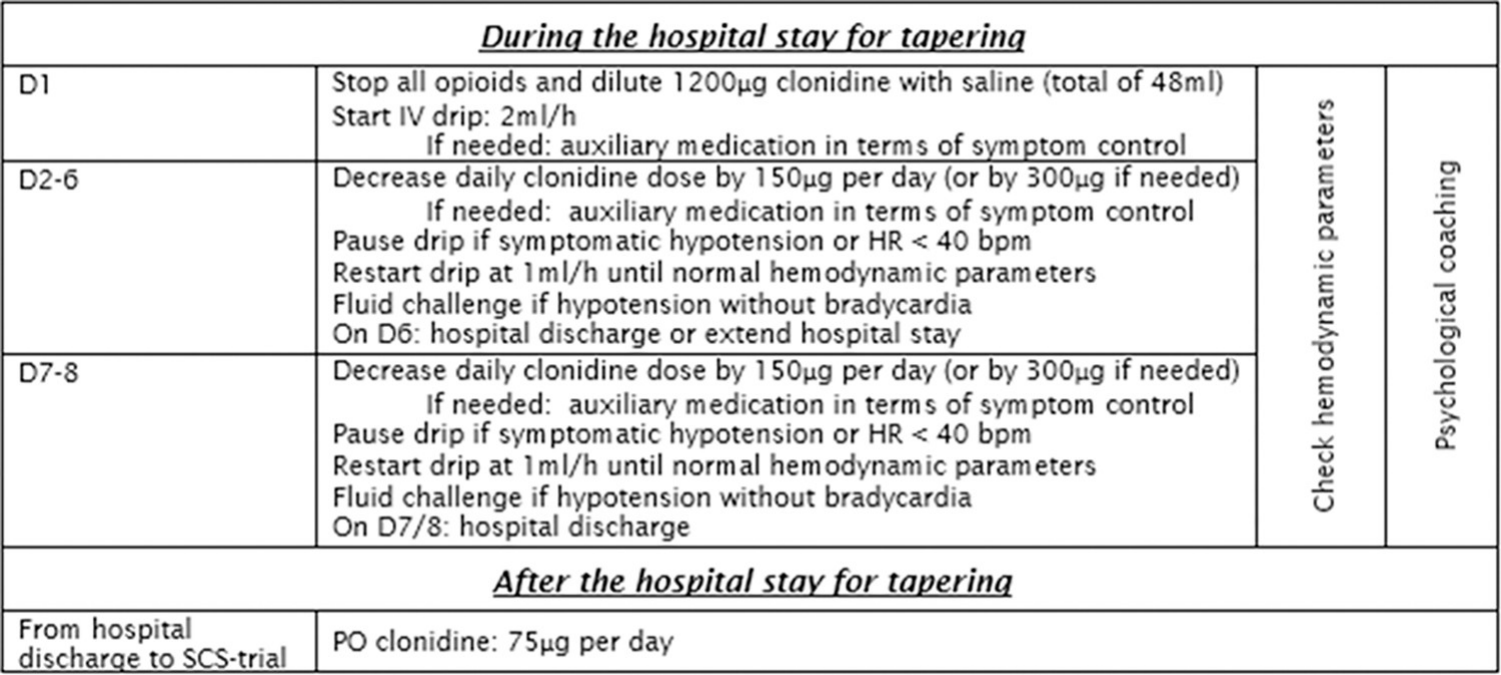
Detailed overview of the standardized pain medication tapering program. Abbreviations. D: Day; IV: Intravenous; HR: Heart rate; PO: Per os.

For each patient within the standardized tapering group (N = 65), a standardized protocol will be delivered to wean off opioids and gabapentinoids. A schematic overview of this tapering protocol is presented in Fig 3.

### In- hospital stay

At admission, all opioids and gabapentinoids (if taken) will be stopped, and clonidine diluted in saline will be administered to counteract withdrawal symptoms with a rate of 2 mL/h to reach a maximum dose of 1200μg/48mL. Over the following hospital days, clonidine will be decreased with 150μg per day until the end of hospitalization after six (or maximum eight) days. Throughout the protocol, auxiliary medication including pain medication, is provided as needed in terms of symptom control. Blood pressure and heart rate will be monitored during the entire hospital stay. The drip of clonidine will be temporarily lowered to a rate of 1mL/h in case of a heart rate between 40 and 45 bpm. In case of symptomatic hypotension or a heart rate lower than 40 bpm, clonidine will be stopped. In the case of symptomatic hypotension without bradycardia, a fluid challenge bolus of 250 mL will be administered, with the possibility of an additional 250 mL of saline. This protocol has been implemented and is published by the principal investigator [28]. Patients will be asked to complete the VAS-pain and the VAS for opioid craving at hospital discharge.

### After the hospital stay

After the hospital stay, all patients will continue to use oral clonidine (75μg per day) up to the SCS trial.

### Experimental intervention: Personalized tapering protocol

**Fig 4:**
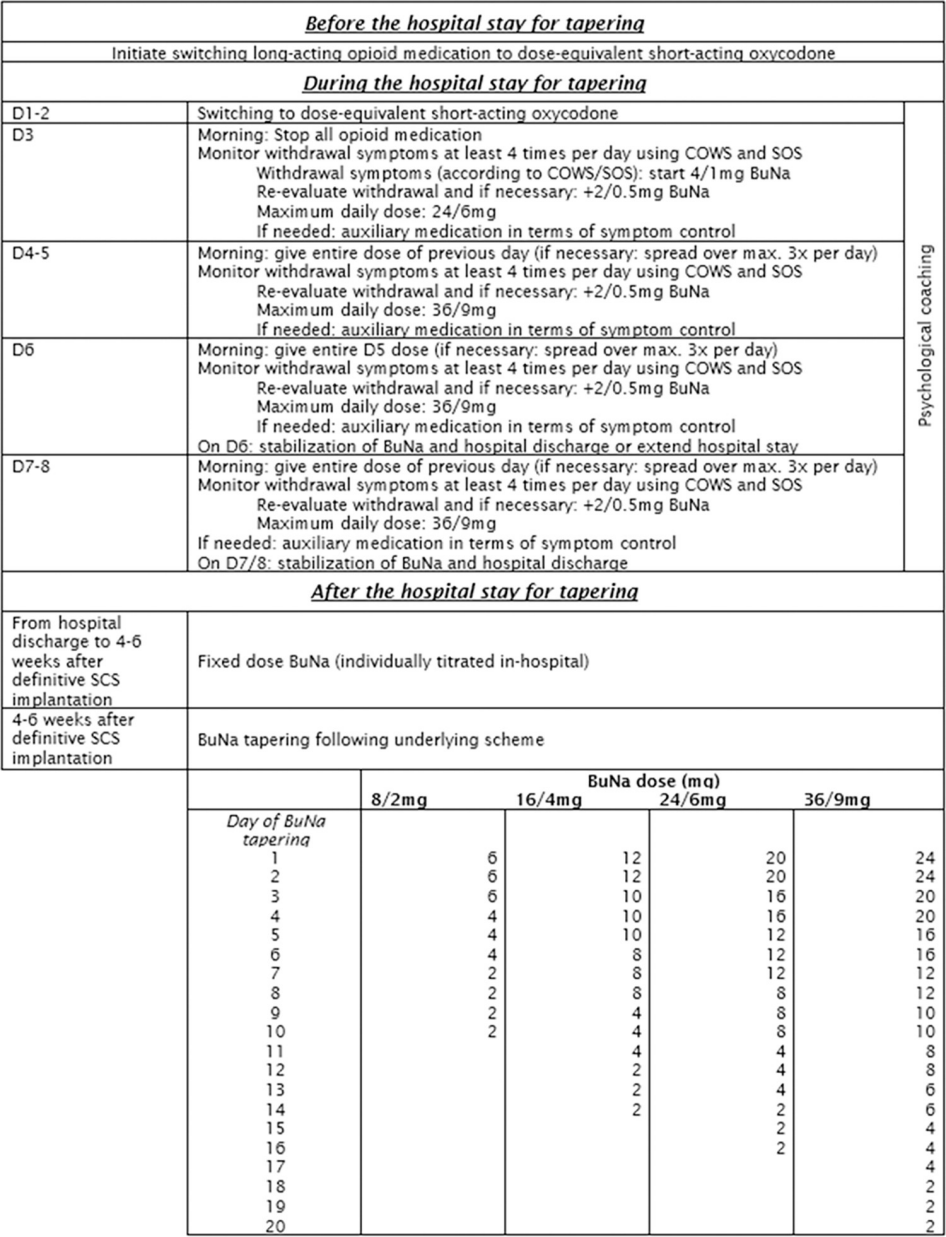
Detailed overview of the personalized pain medication tapering program. Abbreviations. D: Day; COWS: Clinical Opiate Withdrawal Scale; SOS: Subjective Opioid Scale; BuNa: Buprenorphine/Naloxone.

Patients randomized to the personalized tapering protocol (N = 65), will be converted to the partial opioid agonist Buprenorphine/Naloxone (BUNa) before initiating neuromodulation. Patients will be hospitalized for six to eight days to induce them on BuNa. As of hospital discharge, patients will remain on a stable BuNa dose for four to six weeks after definitive SCS implantation, after which BuNa will be tapered off. A schematic overview of this tapering protocol is presented in Fig 4.

### Before the hospital stay

If necessary, patients will start with a substitution of long-acting opioids to short-acting oxycodone at home. Per patient, an individual medication scheme will be provided, based on their current medication usage. With the aid of opioid conversion tables, the conversion to short-acting oxycodone is made, whereby the converted dose will be reduced by 25%. The maximum dose thus depends on the daily dose taken by the patient, with a reduction of 25%. For patients who only use short-acting opioids, no substitution is needed prior to hospital admission.

### In- hospital stay

During the first two days, if necessary, substitution to oxycodone will be initiated and assessed, or continued when patients started the substitution at home. On the morning of the third hospital day, oxycodone is stopped and BuNa is initiated sublingually as of the appearance of withdrawal symptoms (as described by Ellerbroek et al. [33]). For BuNa induction purposes, pain, craving and withdrawal are evaluated at least 4 times per day using the Subjective Opioid Scale (SOS), the Clinical Opiate Withdrawal Scale (COWS), the VAS pain, and VAS craving. Upon withdrawal symptoms (assessed as a score >13 on COWS and corroborated by positive SOS-score), BuNa is provided at a starting dose of 4/1mg BuNa and can be increased by 2/0.5mg as needed until a maximum dose of 24/6mg has been reached. On the morning of day 4 and 5 of the hospital admission, patients receive the entire dosage of BuNa from the previous day, and withdrawal symptoms are further assessed. If needed, additional BuNa is given by increments of 2/0.5mg with a maximum dose of 36/9mg. On the morning of day 6, patients receive the entire dosage of BuNa from the previous day. On this final day of hospitalization, a stable dose is evaluated, and minor changes are possible (minor dose reduction, minor dose increases, or dose breaking up to three times daily). Dosing will be increased if the patient experiences withdrawal and decreased if the patient experiences drowsiness. If the hospital stay is prolonged to eight days, BuNa dosing will be similar to days four, five and six until a stable dose is reached. Throughout the protocol, auxiliary medication including pain medication, is provided as needed in terms of symptom control. This protocol has been used in non-cancer chronic pain patients with good results on pain relief and craving [32]. Patients will be asked to complete the VAS-pain and the VAS for opioid craving at hospital discharge.

### After the hospital stay

After the hospital stay, patients remain on a fixed dose of BuNa until 4–6 weeks after IPG implantation, followed by the tapering of BuNa, as shown in Fig 4. Gabapentinoids (if taken) will be tapered after IPG implantation during a 2-week period, at a rate of 50% per week.

### Both tapering programs

The hospital pharmacy of each participating center will be responsible for storage, labelling and delivery of Clonidine, short-acting Oxycodone and BuNa. An outpatient tapering program alternative will be available in case patients use low opioid dosages. The medical team at each cooperating hospital will be trained in performing the tapering programs by members of the PIANISSIMO consortium. Once the trial is initiated, the medical teams will be able to contact the PIANISSIMO consortium directly, allowing them to discuss any difficulties experienced on short notice.

Psychological coaching is provided to patients in both pain medication tapering arms, using educational videos in which an AI generated avatar explains key concepts during the tapering. These videos are provided on a daily basis during the hospital stay to increase motivation and persistence during the tapering, and to offer tools to prevent relapse and to cope with pain after the hospital stay [34,35]. During the first day, patients will receive 3 videos about pain neuroscience education in which the physiology of the nervous system and the pain system is explained, including changes during sensitization and chronic pain and the mechanisms of action of SCS. On the second day, a detailed explanation about several types of pain medication, namely morphine, gabapentinoids, and benzodiazepines is provided (2 videos). During the third day, 3 videos elaborate on breaking through habits including patient testimonials. On the fourth day, patients receive educational content about problem solving and coping strategies (2 videos). On the fifth day, patients will learn relapse strategies and prevention techniques (2 videos). Patients may watch these videos several times and can also discuss them with family members, if preferred.

### Strategies to improve adherence to interventions

In case patients were allocated to a pain medication tapering program, residual study medication will be collected at the follow-up assessment at 3 months, as part of the quality assessment (compliance to the study protocol). The medication will then be given to the pharmacy of the Universitair Ziekenhuis Brussel for destruction.

At fixed time points (upon admission for SCS trial implantation, at 1 month, 3 months, 6 months, and 12 months after IPG implantation) a urine sample is collected. This sample will be analyzed (by urine dipsticks for opioids, benzodiazepines, gabapentin and pregabalin) at the time of collection and will be destroyed afterwards. With these urine dipsticks, we can objectively evaluate usage of opioids, benzodiazepines, gabapentin and pregabalin.

### Outcomes

#### Primary outcome measure: Disability

Functional disability due to low back pain will be assessed by the Oswestry Disability Index (ODI) [36,37]. The ODI contains ten topics: pain intensity, lifting, ability to care for oneself, ability to walk, ability to sit, sexual function, ability to stand, social life, sleep quality and ability to travel. Each topic is scored on a Likert scale from 0 (no disability) to 5 (maximum disability). The scores for all questions are summed and multiplied by two to obtain the index score (range 0–100), with high scores representing high disability. In patients with PSPS-T2, the minimum clinically significant difference in ODI score is 9.0 with a sensitivity of 0.74 and a specificity of 0.92 [38].

#### Secondary outcome measurements

*Pain intensity*. Current pain intensity and mean pain intensity during the past 7 days will be assessed using the Visual Analogue Scale (VAS—100 mm), separately for leg and low back pain. The VAS pain score is a reliable measure, being valid and sensitive to change [39,40]. The VAS has a good test-retest reliability [41].

*Health-related quality of life*. To describe health-related quality of life, the EuroQol with five dimensions and five levels (EQ-5D-5L) will be used [42]. Dimensions include mobility, self-care, usual activities, pain/discomfort, and anxiety/depression, with five response levels per dimension. The scores from the five dimensions are converted into an index value, with a range from <0 (where zero is a health state equivalent to death; negative values are valued as worse than death) to 1 (perfect health). The EQ-5D-5L has been validated in patients with low back pain [43,44] and a Belgian EQ-5D-5L value set is available [45].

*Participation*. The Impact on Participation and Autonomy Questionnaire (IPA) will be used to measure participation and autonomy [46,47]. This questionnaire provides a measure of limitations in participation and autonomy and includes 39 questions across 5 domains: autonomy indoors, autonomy outdoors, family role, social life and relationships, and work and education. Participants rate each item on a Likert scale from 0 (very good) to 4 (very poor). The scoring captures how likely respondents feel they will be able to participate in a described activity and how their disability impacts their ability to participate. Higher scores indicate more important restrictions. The IPA has good psychometric properties, including reliability, validity, and responsiveness to intervention [47,48].

*Domains affected by substance use*. To evaluate domains affected by substance use, 3 outcome measurements will be used. The Measurements in the Addictions for Triage and Evaluation (MATE) is a structured interview used to assess drug (including opioid) related patient characteristics and associated difficulties [49]. It evaluates the use of psychoactive substances, history of substance use treatment, and substance craving [49]. In addition, it establishes social engagement, identifies contextual influences on engagement and the resulting need for treatment [49]. To measure severity of craving, a VAS for opioid craving (OCVAS) is used [50]. The Current Opioid Misuse Measure (COMM) is a 17-item questionnaire that assesses the risk for aberrant medication-related behavior. Each question is graded from 0 (never) to 4 (very often) resulting in a total score between 0 and 68. The questionnaire is a reliable and valid tool in patients with chronic pain who are prescribed opioids for their pain [51].

*Medication usage*. The Medication Quantification Scale III (MQS III) and later versions will be used to quantify pain medication regimens in a wide variety of pain conditions [52]. It provides a numerical output that represents the negative impact of each medication [53]. For each medication, a MQS score is calculated by multiplying a detriment weight for a given pharmacologic class with a score for dosage [54]. Medication is subdivided into five classes: non-steroidal anti-inflammatory drugs, muscle relaxants, neuropathic pain medications (antidepressants and anticonvulsants), benzodiazepines and opioids. All calculated values are summed to obtain a total MQS score.

*Healthcare expenditure and indirect costs*. Healthcare expenditure will be investigated by two means. For the expenditures associated with in-hospital care, data will be extracted from hospital claims data. The other costs are collected through self-reporting methods, for which a combination of weekly self-reports with a diary (from baseline to IPG implantation) and questionnaires (follow-up assessments) will be used. Absence from work (indirect costs) is documented via questionnaires and valued using the human capital approach. Hence, healthcare expenditure includes the number of days spent in hospital, medical tests related to surgery and any kind of post-surgical treatments (e.g., pain medication, physiotherapy).

Psychological constructs

(I) The Hospital Anxiety and Depression Scale (HADS) aims to measure symptoms of anxiety and depression and consists of 14 items: seven items for the anxiety subscale (HADS Anxiety) and seven for the depression subscale (HADS Depression). Each item is scored on a response-scale with four alternatives ranging between 0 and 3. After adjusting for six items that are reversed scored, all responses are summed to obtain the two subscales. Recommended cut-off scores are 8–10 for doubtful cases and ≥ 11 for definite cases [55]. The HADS was found to perform well in assessing the symptom severity of anxiety disorders and depression in both somatic, psychiatric, and primary care patients and in the general population [56].
(II) The General Self-Efficacy (GSE) scale is a 10-item self-report questionnaire and will be used to assess perceived self-efficacy. Self-efficacy is the belief in one’s competence to cope with challenging demands and the belief in one’s capabilities to achieve goals [57–59]. Self-efficacy beliefs determine whether coping behavior is initiated, how much effort is expended, and how long this behavior is sustained in the face of obstacles and aversive experiences [60].
(III) The Pain Catastrophizing Scale (PCS) is a self-reported questionnaire used to assess catastrophic thoughts or feelings accompanying the previously experienced pain [61,62]. It consists of 13 items that evaluate 3 subscales of catastrophizing: rumination, magnification and helplessness on a 5-point Likert scale [61,62]. The PCS factor scales are valid and reliable in patients with chronic pain [61–63].
(IV) The Multidimensional Pain Inventory (MPI), a 61-item questionnaire, will be used to assess the coping strategy profile. This profile is created on the behavioral and psychological responses to the chronic pain experience. The MPI is divided in three parts to evaluate the pain experience, the responses of significant others to communications of the patients’ pain, and the patients’ report of participation in daily activities. The MPI is valid in patients with chronic pain eligible for SCS [64].

#### Sleep quality

The Pittsburgh Sleep Quality Index (PSQI) is a self-rated questionnaire used to measure sleep quality during the last month [65,66]. It includes 24 items and 7 component scores: subjective sleep quality, sleep latency, sleep duration, habitual sleep efficiency, sleep disturbances, use of sleeping medication, and daytime dysfunction [66]. It is a reliable and valid measurement tool with good internal consistency and construct validity in patients with chronic pain [67].

*Symptoms of central sensitization*. The Central Sensitization Inventory (CSI) will be used to assess symptoms associated with central sensitization [68]. The total score of the CSI ranges between 0–100. The CSI has good psychometric properties including initial construct validity, test-retest reliability, good discriminative power, and good internal consistency in patients with chronic pain [69].

**Fig 5:**
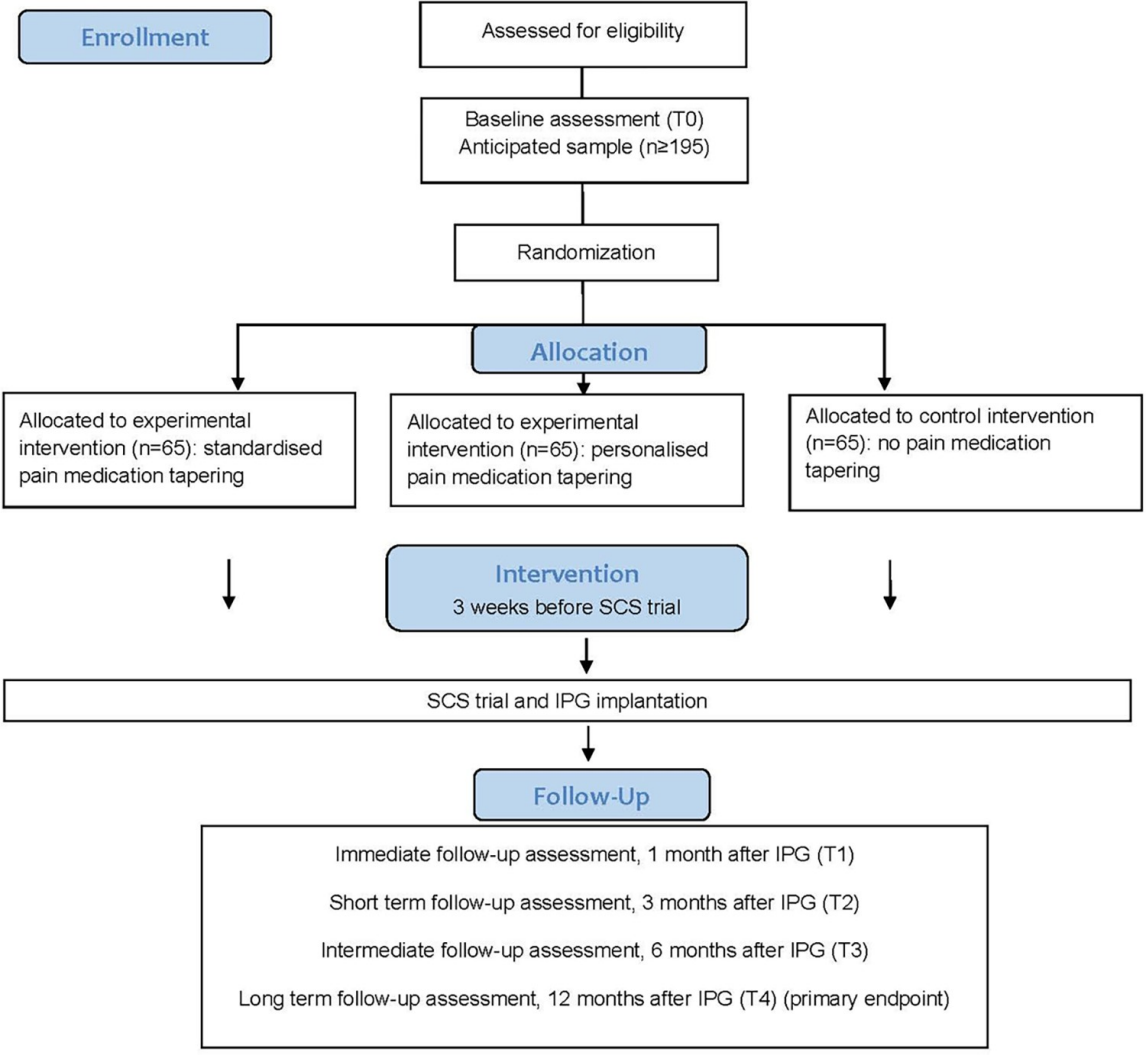
Project flowchart. Participant timeline. Abbreviations. IPG: Implantable pulse generator, n: Number, SCS: Spinal Cord Stimulation, T: Time.

At baseline, patients are asked about their demographics and socio-economic status (sex, age, marital status, years of education, educational level, employment status, occupation, income, and household members) and baseline clinical data (number of previous surgeries, duration of pain, how many years they have used pain medication). In addition, a retrospective questionnaire about their healthcare usage over the period of three months prior to the baseline assessment is administered. The Mini International Neuropsychiatric Interview (MINI) will be administered in order to determine major psychiatric comorbidities [70]. Patients are seen by the investigator at one month, three months, six months, and twelve months after IPG implantation to complete the follow-up assessments according to the study protocol. Fig 5 presents the project flowchart.

#### Sample size

The sample size calculation was performed on the primary outcome measure, i.e., “disability, measured with the ODI”, at 12 months. The following assumptions were made:

- The mean disability score at 12 months with SCS is 33.34 (SD: 16.86) [71].
- The expected difference between tapering and no tapering at 12 months of SCS is 19.64% [24], leading to an expected total ODI score of 26.79 in the tapering groups.
- A 10% decrease in ODI score is assumed with the personalized tapering protocol compared to a standardized tapering protocol (assumption since no ODI data is available to estimate the actual beneficial effect).
- The common standard deviation (16.789) is based upon 4 repeated measures from an earlier trial in an identical study population [71].

To conduct a three-arm parallel group, randomized clinical trial with 5 assessments, and estimated true mean ODI responses for the no tapering group of 33.34, standardized tapering group of 26.79 and personalized tapering group of 24.11, with a common standard deviation of 16.789, and 80% power to detect differences in means at a 5% two-sided significance level with Bonferroni correction, a total sample size of 147 patients is needed. This calculation was performed by the guidelines provided in the book of Chow et al. concerning sample size calculations in clinical research [72] and controlled with G power version 3.1. To account for a SCS trial failure rate of 11.3% [71] and a general loss-to-follow-up of 20%, a sample size of 65 patients per arm will be needed. This results in a total sample size of 195 patients for this study.

#### Recruitment

Patient recruitment will take place in three centers in Belgium: Universitair Ziekenhuis Brussel, Heilig Hart Ziekenhuis Lier, and AZ Delta. Patients are recruited from September 29, 2023 onwards and recruitment will continue until the predetermined number of patients in included. Depending on the rate of inclusion, other centers will be contacted as well. Treating neurosurgeons or anesthesiologists (regional coordinating investigator or his/her designee) will inform eligible patients about the project when patients are scheduled for a treatment trajectory with SCS.

### Allocation

#### Sequence generation

Randomization occurs by computer-generated random number sequence (Sealed Envelope Ltd., available from: https://www.sealedenvelope.com/simple-randomiser/v1/lists [Accessed 4 July 2023]). A 1:1:1 allocation will be used. Stratified randomization will be used according to investigational site and pain medication usage with 4 categories based on 1) opioid usage ≥ 90 MME/day or below and 2) pregabalin > 600 mg/day or gabapentin > 1800 mg /day.

#### Concealment mechanism

An independent researcher of the PIANISSIMO consortium has direct access to the randomization list (one list for all centers). This researcher provides the randomization allocation to an unblinded PhD researcher who will arrange practical issues such as appointment times with the hospital wards to perform the experimental interventions. The randomization list is inaccessible for the outcome assessor. The independent researcher who has access to the randomization will not perform interventions, nor the measurements.

### Implementation

The unblinded PhD researcher is responsible for informing the patients into which group they are randomized and for scheduling the hospital stay, if the patient is allocated to one of the pain medication tapering arms.

### Blinding

The statistician and outcome assessors will be blinded to group allocation. Patients cannot be blinded as a hospital stay of at least six days is necessary in both intervention arms. Patients will be asked not to communicate with the outcome assessors about the intervention received. Furthermore, at the end of each assessment, the success of assessor blinding will be examined by asking whether the assessor thought the patient had received one of the experimental interventions or control intervention, including the percentage of certainty. Neither the statistician nor the outcome assessors will be unblinded during the trial.

### Data collection and management

To optimize the study feasibility and to ensure data protection, the self-reported measures will be completed online using Qualtrics, in the hospital setting. After inclusion, patients will complete the baseline assessment, assessing socio-demographic information, baseline clinical data, MINI neuropsychiatric interview and questionnaires related to our primary and secondary outcome measures. Additionally, they will receive a diary for healthcare utilization that will be completed until IPG implantation.

*Data management*. Data will be collected via web-based self-reported questionnaires that are provided to the patients on a tablet in the hospital. An error message will appear when a question is not filled in, to avoid missing data. Collected data will include answers to validated questionnaires related to the participants’ disability, pain intensity, health-related quality of life, participation, domains affected by substance use, anxiety and depression, medication usage, psychological constructs, sleep, and symptoms of central sensitization. Healthcare expenditure will be reported through weekly self-report on paper until IPG implantation, and with web-based questionnaires afterwards. In addition, participants will be asked about the number of previous surgeries, the duration of how long they are experiencing pain, how many years they have used pain medication and general socio-demographic data (e.g., sex, age, marital status, years of education, educational level, employment status, occupation, income, and household members). Further, the patients’ name, telephone number, e-mail, and hospital claims will be collected. Results of the urine dipsticks for opioids, benzodiazepines, gabapentin and pregabalin (collected before SCS trial, and at 1 month, 3 months, 6 months and 12 months after IPG implantation) will be stored electronically, after double verification by the outcome assessor. Urine samples and dipsticks will be destroyed immediately after data collection. Results of the VAS-pain, OCVAS, SOS and COWS which are collected at least 4 times per day during the personalized tapering program, will be collected on paper. Manual conversion of data towards electronic format will be conducted by two independent reviewers (unblinded researchers) to ensure data quality. At hospital discharge, the VAS-pain and OCVAS will be assessed in both tapering groups through web-based data collection. Safety information, through monitoring of (serious) adverse events, will be collected on paper. Self-written codes in R and SAS will be created to analyze the data. Written informed consent of the patients will be collected and provides the basis for the legal ground for the data management.

During the research, the persons responsible for data management and storage will be the PhD investigators. Following the research, the principal investigator will be fully responsible for data management and storage. During the research, all obtained data will be stored on a dedicated page on Vrije Universiteit Brussel SharePoint (system-encrypted) with access limited to the investigators and supervisors. A back-up will be foreseen on a secured external hard drive. Following the research, all data will be relocated to the Vrije Universiteit Brussel Archive where it will be archived for 25 years. All possible personal identifiable data (vide infra) will be removed from the archived data.

Personal data will be processed in accordance with the ongoing European Union’s Data Protection Directive and regulation, the relevant Belgian legislation concerning data protection of July 30^th^, 2018, and good clinical practice. As we collect personal identifiable data, following steps are taken to limit unauthorized access. Informed consents will be preserved at a secure location at the Vrije Universiteit Brussel. Qualtrics (Qualtrics, Provo, UT) will be used for data collection to improve data protection as responses to questionnaires will only be accessible to the investigator. Collected data will be password protected. Personal identifiable and clinical trial data will be separated, with the latter receiving a unique participant ID. Access to informed consents, personal identifiable data and the link with the participant ID will be restricted to the investigators and supervisors and stored separately from the trial data. Eventual further dissemination of data will only occur in a pseudonymized or aggregated way.

The Vrije Universiteit Brussel supports the FOSB metadata standard (= dataset metadata schema defined by the Flemish Open Science Board) which can be mapped to the international DataCite metadata schema. At the project level, the general information (title, investigators, aim, objectives, concepts, hypotheses, funder), protocol, sampling procedure, instruments, hardware and software used to collect data, data handling log, accessibility of the data, and data manipulations will be provided in research plans, and publications. At the database level, an inventory of the files will be provided in a read-me file. At the data level, a codebook will be provided on how to handle quantitative variables together with the scripts to analyze the data.

### Confidentiality

Participant identification codes will be used to link data to patients. The file containing the link between participant numbers and personal data (i.e. key) will be managed by the researchers and will be locked for access by others. As an additional security measure, the file linking the pseudonymization to the original direct identifiers will be encrypted before it is uploaded to SharePoint.

### Statistical methods

Longitudinal mixed model analysis will be used to evaluate and compare therapy effects. The need for random intercepts and slopes will be evaluated. Potential confounding variables (e.g., baseline medication use) will be considered in the analysis. Statistical, as well as clinically significant differences will be defined at alpha < 0.05. Furthermore, based on the baseline data, we will determine predictive factors and which subgroup of patients will benefit the most of a tapering program before initiating SCS, by several machine learning techniques (both supervised and unsupervised techniques). No interim analyses will be conducted as we do not foresee any potentially serious outcomes.

### Methods for additional analyses (e.g. subgroup analyses)

Baseline data will provide cross-sectional results on demographics, socio-economics, baseline clinical data, and outcome measurements for the complete PSPS-T2 group and comparisons between possible subgroups. Correlation analyses will be performed to unravel correlations between the different outcome measurements in patients with PSPS-T2, eligible for SCS.

Additionally, a within trial economic evaluation will be conducted [73] on the intention to treat population. All costs of all participants will be considered, starting from three months before baseline assessment until the end of the 12-month follow-up period. The valuation of resource use is based on national tariffs. A societal perspective is adopted. Health outcomes will be expressed in two ways. Effects are expressed in percentage disability, which is the primary outcome in this trial. Next, health outcomes will be considered, expressed in utility using health state values from the general public, in accordance to the Belgian guidelines [74]. The comparator is the control group receiving no pain medication tapering. Differences in cost between the treatment arms will be analyzed using generalized linear models. Modified Park test will be used to identify the appropriate link function [75]. The overall result is expressed in an incremental cost effectiveness ratio. The point estimates of incremental costs and increment health benefits (deterministic analyses) are subject to uncertainty which will be addressed in probabilistic analyses [75]. We will apply nonparametric bootstrapping to test for statistical differences in costs and health benefits to investigate the uncertainty around these outcomes and summarized in cost-effectiveness acceptability curves indicating the likelihood of the intervention to be cost-effective at any willingness-to-pay threshold. Reporting on the results of the health economic evaluation will be in line with the CHEERS-guidelines [76]. Besides the within-trial health economic evaluation, a model-based evaluation will be conducted to estimate the expected costs and health outcomes in both the control and intervention arms beyond the follow-up period of the trial. A Markov-model will be developed compliant to the commonly used guidelines. We assume a cycle of 1 year in the model and apply a lifetime horizon. Lifetime incremental costs and quality-adjusted life years will be the input for the ICER calculation. Discount rates of 3% for costs and 1.5% for utilities will be applied, which is in line with the Belgian guidelines [77]. The subsequent probabilistic analyses and reporting strategies are identical to those described above.

Finally, a nested process evaluation will be conducted in parallel to this RCT.

### Methods in analysis to handle protocol non-adherence and any statistical methods to handle missing data

Both intention-to-treat as well as per protocol analyses will be conducted to check whether both definitions of the population will point towards similar results, which could demonstrate robustness of the results. At first, analysis will be performed on data as observed, since random effects mixed models enable us to conduct the analysis in case one valid measurement is included in the analysis. Hence, intermittent missing is not expected to substantially bias the results. To address possible informative drop-outs, a sensitivity analysis will be performed using multiple imputation, including all available information on background characteristics and outcomes.

### Plans to give access to the full protocol, participant level-data and statistical code

After finalizing the project, access restrictions will be applied to the pseudonymized data and will be specified in a data use agreement containing following elements: evaluation of the re-use request by the ethical committee, non-disclosure agreement and warranties for safely storage of data.

### Oversight and monitoring

#### Composition of the coordinating centre and trial steering committee

The Steering Board is the main decision-making and steering body of the project. The Steering Board consists of M.M, C.L.C., K.P., and L.G. The extended Steering Board consists of the Steering Board, together with the project coordinator and two PhD students. The Steering Board organized a kick-off meeting at the start of the project to establish common working procedures. The main tasks of the Steering Board are: 1) agree on common working procedures and management policies; 2) monitor overall progress and follow-up of deliverables; 3) decisions on major changes to the work program; 4) conflict handling; and 5) budget decisions. One of the most important tasks of the Steering Board is also to (re)direct all participants in the implementation of the work program and the timely achievement of all deliverables. The Extended Steering Board will further assemble meetings at least every six months. Additional teleconferences can be organized ad hoc in case of urgent issues. The Extended Steering Board is responsible for assuring the quality of the workflow and project implementation, considering the available resources. The Principal Investigators in the recruiting centers are responsible for patient recruitment and form the PIANISSIMO consortium, together with the Extended Steering Board.

The valorisation board consists of relevant stakeholders (N = 10) who will be asked to actively contribute to implementation of the study findings, and any difficulties experienced during the implementation process will be discussed. The researchers are available to support the stakeholders with the implementation process. In addition, the valorisation board will prepare and guide the full utilisation process in the period following the completion of the research project (after-trajectory), together with the Extended Steering Board.

#### Composition of the data monitoring committee, its role and reporting structure

The study coordinator at Vrije Universiteit Brussel will regularly monitor data that are entered electronically. The study coordinator is independent from the funder of this study and has no competing interests.

### Adverse event reporting and harms

All adverse events reported spontaneously by the patient or observed by the assessor will be recorded. Serious adverse events will be reported to the (local) principal investigators as soon as possible, who will be responsible for informing the ethics committee. Suspected Unexpected Serious Adverse Reactions will be reported to the ethics committee as soon as possible.

### Frequency and plans for auditing trial conduct

The study staff will submit a summary of the progress of the trial to the central ethics committee once a year. Information will be provided on the date of inclusion of the first subject, number of subjects included, and number of subjects that have completed the trial, serious adverse events/ serious adverse reactions, other problems, and amendments. Additionally, there is planned on-site auditing of the trial by the Clinical Trial Center of Universitair Ziekenhuis Brussel. To ensure compliance with relevant regulations, an independent quality assurance representative may review this study. This implies that auditors will have the right to audit the site(s) at any time during and/or after completion of the study and will have access to the data generated during the clinical investigation, source documents, and patient’s files.

### Plans for communicating important protocol amendments to relevant parties (e.g. trial participants, ethical committees)

All protocol amendments will need to be approved of by the ethics committee prior to implementation. If relevant, patients will be informed of protocol modifications.

### Dissemination plans

All stakeholders will be contacted during the execution of the project to start introducing the tapering approach before neuromodulation, in patients with PSPS-T2. Regular updates will be provided to all stakeholders to keep everyone informed, involved, and motivated for this project. Further, we will communicate findings of this project via the publication of scientific manuscripts and presentations on national and international symposia, as well as through social media (STIMULUS & UMCOR social media accounts). Furthermore, we will write a summary of the main study findings in layman’s terms for patients’ organizations and charities. Additionally, social instances will be contacted, and presentations will be given to make them aware of the study findings.

## Discussion

Long-term use of opioids and gabapentinoids is associated with adverse events such as drowsiness, headaches, nausea or confusion, the risk of opioid-induced hyperalgesia and increased risks for tolerance and both physical and psychological dependence [11,78–80]. Despite the CDC guidelines [81] and the well-known risks of long term use of medication, pain medication tapering programs, or the consideration of whether these programs are necessary, are not yet part of the standard treatment trajectory of patients with prolonged pain medication usage. Presumably, the large heterogeneity in proposed programs (e.g., fast versus slow tapering, mono- or multidisciplinary programs, in-hospital versus at home tapering, type of pharmacological agents) is one of the underlying reasons for the lack of implementation in clinical practice [82,83]. In this study, we aim to evaluate whether disability is altered after patients undergo a standardized, personalized or no pain medication tapering program before SCS. If pain medication tapering programs are deemed to be more effective than no tapering, this would add to the evidence towards an improved patient-centered care model in this patient group and set a clear path to advocate for pain medication tapering as the new standard treatment guideline for these patients before SCS.

## Trial status

Recruitment has started in September 2023 and will be ongoing until 195 patients are included in the study (expected end date September 2025). The current protocol is version 2 of April 2023.

## Supporting information

S1 Checklist SPIRIT 2013 checklist: Recommended items to address in a clinical trial protocol and related documents*. (PDF)

S1 File (PDF)

## Abbreviations

BuNa: Buprenorphine/Naloxone
COWS: Clinical Opiate Withdrawal Scale
CSI: Central Sensitization Inventory
EQ-5D: 5l: EuroQol with five dimensions and five levels
GSE: General Self-Efficacy
HADS: The Hospital Anxiety and Depression Scale
IPA: Impact on Participation and Autonomy Questionnaire
IPG: implantable pulse generator
MATE: Measurements in the Addictions for Triage and Evaluation
MINI: Mini International Neuropsychiatric Interview
MME: morphine milligram equivalents
MPI: Multidimensional Pain Inventory
MQS III: Medication Quantification Scale III
OCVAS: VAS for opioid craving
ODI: Oswestry Disability Index
PCS: Pain Catastrophizing Scale
PSPS-T2: Persistent Spinal Pain Syndrome Type II
PSQI: Pittsburgh Sleep Quality Index
SOS: Subjective Opioid Scale
VAS: Visual Analogue Scale
