# Supplementary material for: Pain medication tapering for patients with Persistent Spinal Pain Syndrome Type II, treated with Spinal Cord Stimulation: A RCT–study protocol of the PIANISSIMO study
Source: PLoS One. 2024 Aug 12;19(8):e0302842. doi: 10.1371/journal.pone.0302842 (PMC11318931; doi:10.1371/journal.pone.0302842)
Supplement: S1 File — (PDF) [file pone.0302842.s002.pdf]

Study Protocol

Study Title: *Pain medication tapering for patients with Persistent Spinal Pain Syndrome Type II, treated with Spinal Cord Stimulation*
Study Acronym: *PIANISSIMO*
Phase of Development: *NA*
Protocol Number: *1*
Protocol Version and Date: *Version 2, April 2023*
EudraCT Registry Number: *2022-003925-23*
Indication: *Pain medication tapering in Persisting Spinal Pain Syndrome Type II*
Investigational products*: Clonidine, Oxycodone, Buprenorphine/Naloxone*
Sponsor: *Vrije Universiteit Brussel*
Coordinating/Principal Investigator: *Prof. Dr. Maarten Moens*

PROTOCOL SIGNATURE PAGE

Protocol Version and date: *Version 2, April 2023*

Protocol Title: *Pain medication tapering for patients with Persisting Spinal Pain Syndrome Type II, treated with Spinal Cord Stimulation*

Sponsor: *Vrije Universiteit Brussel*

Principal Investigator: *Prof. Dr. Maarten Moens*

I agree:
- *to assume responsibility for the proper conduct of this study*
- *to conduct the study in compliance with this protocol and any future amendments*
- *not to implement any deviations from or changes to the protocol without prior review and written approval from the Ethics Committee, except where necessary to eliminate an immediate hazard to the participants, or for administrative aspects of the study (where permitted by all applicable regulatory requirements)*
- *that I am thoroughly familiar with the appropriate use of the investigational drug, as described in this protocol*
- *to ensure that all persons assisting me with the study are adequately informed about the investigational drug and their study-related duties and functions as described in the protocol*
- *that I am aware of and will comply with the current good clinical practice (GCP) guidelines and ethical principles outlined in the Declaration of Helsinki*
- *to conduct the study in accordance with all applicable laws and regulations*

Table of Contents

5.1 Study Design ... 9

5.2 Research methodology ... 9

5.3 Patient recruitment ... 9

5.4 Baseline assessments ... 10

5.5 Outcome measures ... 10

5.6 Early study termination ... 12

5.7 End of study ... 12

5.8 Sample size calculation ... 12

6.1 Primary Objective ... 13

6.2 Secondary Objectives ... 13

6.3 Endpoints ... 13

7.1 Inclusion Criteria ... 13

7.2 Exclusion Criteria ... 13

8.1 Screening and Enrollment ... 14

8.2 Randomization ... 14

8.3 Blinding Procedures ... 14

9.1 Treatments Administered ... 14
9.1.1 Control intervention: no tapering ... 14
9.1.2 Experimental Intervention: standardized tapering protocol ... 14
9.1.3 Experimental Intervention: personalized tapering protocol ... 15

9.2 Summary of Product Characteristics ... 16

9.3 Direction of administration and dosing regimen ... 16

9.4 Storage, Labeling and Delivery ... 16

9.5 Study Disposal and Destruction ... 16

10.1 Screening ... 18

10.2 Baseline ... 18

10.3 Treatment period: hospital stay ... 18

10.4 SCS trial and definitive SCS implantation ... 18

10.5 Follow-up assessments ... 18

10.6 Assessment Types ... *18*

11.1 Adverse Events and Adverse Reactions ... *18*
11.1.1 Definitions and Reporting ... 18
11.1.2 Reporting Period ... 19
11.1.3 Intensity ... 19
11.1.4 Relationship to treatment ... 19

11.2 Serious Adverse Event or Reaction ... *19*
11.2.1 Definitions ... 19
11.2.2 Immediate Reporting ... 20

11.3 Suspected Unexpected Serious Adverse Reaction (SUSAR) ... *20*
11.3.1 Definitions ... 20
11.3.2 Reporting ... 20

11.4 Other safety data requiring an immediate declaration ... *20*

11.5 Procedures for Handling Special Situations ... *20*
11.5.1 Overdose Management ... 20

11.6 Annual Safety Report ... *22*
14.1.1 Baseline analysis ... 23
14.1.2 Main analysis ... 23
14.1.3 Health economic analysis ... 24
14.1.4 Process evaluation ... 24

15.1 Ethical conduct of the study ... *25*

15.2 Informed Consent ... *25*

15.3 Patient and Study Data Protection ... *25*

15.4 Patient Identification ... *26*

17.1 Steering and Advisory Board ... *27*

1 Trial Registration/Protocol Summary

**Information**	
EudraCT number:<br>*Date of registration:*	*2022-003925-23*<br>*06/12/2022*
ClinicalTrials.gov if applicable:	*(Number not yet assigned)*
Official Title:	*Pain medication tapering for patients with Persistent Spinal Pain Syndrome Type II, treated with Spinal Cord Stimulation.*
Study Phase/Type:	*Other*
Condition:	*Persistent Spinal Pain Syndrome Type II (PSPS T2)*
Objectives:	*The* **primary objective** *of the study is to examine whether there is a difference in disability after 12 months of Spinal Cord Stimulation (SCS) in PSPS T2 patients after receiving a standardized pain medication tapering protocol before SCS implantation, a personalized pain medication tapering protocol before SCS implantation, or no tapering protocol before SCS implantation.*<br>*The* **secondary objective** *of the study is to examine whether there is a difference after 12 months of SCS in PSPS T2 patients after receiving a standardized pain medication tapering protocol before SCS implantation, a personalized pain medication tapering protocol before SCS implantation, or no tapering protocol before SCS implantation on pain intensity, health-related quality of life, participation, domains affected by substance use, anxiety and depression, medication use, psychological constructs, sleep, central sensitization, and healthcare expenditure.*
Interventions:	*Pain medication tapering before SCS implantation*
Endpoints:	*The* **primary** *endpoint is to observe whether there is a difference in the* **Oswestry Disability Index** *score in PSPS T2 patients* **12 months after definitive SCS implantation** *between the three arms. A longitudinal mixed model analysis will be used with timepoints defined at baseline, 1 month, 3 months, 6 months, and 12 months after definitive SCS implantation.*<br>*The* **secondary** *endpoint is whether the three arms differ on pain intensity, health-related quality of life, participation, domains affected by substance use, anxiety and depression, medication use, psychological constructs, sleep, central sensitization, and healthcare expenditure after definitive SCS implantation. Longitudinal mixed model analysis will be used with timepoints defined at baseline, 1 month, 3 months, 6 months, and 12 months after definitive SCS implantation.*
Study population:	*PSPS T2 patients scheduled for SCS*
Number of patients:	**Control group (no tapering):** *65 patients*<br>**Standardized pain medication tapering group:** *65 patients*<br>**Personalized pain medication tapering group:** *65 patients*
Overview of study design:	*Multicenter randomized controlled trial*
Sponsor:	*Vrije Universiteit Brussel*<br>*Laarbeeklaan 103, B-1090 Jette, Belgium*
Inclusion Criteria:	- *Patients with PSPS T2, defined as patients suffering from neuropathic pain of radicular origin with pain in the lower back and/or leg(s), of an intensity of at least 4/10 on the Numeric Rating Scale, for a period of at least 6 months after a minimum of one anatomically successful spinal surgery and being refractory to conservative*

	treatment (according to Belgian reimbursement rules from January 1st, 2018)<br>- *Patients need to be scheduled for SCS to be eligible for participation in the study*<br>- *Currently taking opioids*<br>- *18 years and older*<br>- *Speaking and reading Dutch or French*
Exclusion Criteria:	- *Being actively treated for cancer.*<br>- *Having a life expectancy below 6 months.*<br>- *Receiving intrathecal drug delivery.*<br>- *Patients with contraindications for Clonidine (e.g., known hypotension which requires medication) or for Buprenorphine/Naloxone (e.g., severe respiratory insufficiency, hepatic insufficiency).*<br>- *Epilepsy treated by Pregabalin.*<br>- *Currently using benzodiazepines at more than 40 mg diazepam-equivalents per day.*
Target Date of first enrolment:	*June 2023*
Target sample size:	*195*

2 Protocol Version History

Version No.	Release Date	Summary of Changes
1.0	*10/01/2023*	*NA*
2.0	*07/04/2023*	*The removal of Dr. Laurence Leysen as co-investigator and study coordinator from the study team; the removal of Prof. dr. Nick Verhaeghe from the study team; change from 5-day hospital stay for tapering to a 6-day hospital stay, extendable to 8 days if necessary; collection of study medication at 3 months follow-up; adjustment of moments of urine sample; withdrawal symptoms are measured at least 4 times per day instead of every 4 hours.*

3 Sponsor/Coordinating Investigator Information

Sponsor	**Vrije Universiteit Brussel**<br>*Laarbeeklaan 103, B-1090 Jette, Belgium*
Coordinating/Principal Investigator	**Prof. Dr. Maarten Moens**<br>*UZ Brussel - Department of Neurosurgery*<br>*Vrije Universiteit Brussel – Stimulus Research Group*
Co-investigators	**Prof. Dr. Lisa Goudman**<br>*UZ Brussel - Department of Neurosurgery*<br>*Vrije Universiteit Brussel – Stimulus Research Group*<br><br>**Prof. Dr. Koen Putman**<br>*Vrije Universiteit Brussel - Interuniversity Centre for Health Economics Research (I-CHER)*<br><br>**Prof. Dr. Cleo Crunelle**<br>*UZ Brussel - Department of Psychiatry*<br>*Vrije Universiteit Brussel*<br><br>**Dra. Elke Wuyts**<br>*Vrije Universiteit Brussel – Stimulus Research Group*<br><br>**Dra. Frenn Bultinck**<br>*Vrije Universiteit Brussel – Stimulus Research Group and Interuniversity Centre for Health Economics Research (I-CHER)*
Statistician	**Prof. Dr. Lisa Goudman**<br>*UZ Brussel - Department of Neurosurgery*<br>*Vrije Universiteit Brussel – Stimulus Research Group*
Pharmacy if applicable	*Hospital pharmacies of participating sites*
Study sites and co-investigators	**Prof. Dr. Maarten Moens**<br>*UZ Brussel*<br><br>**Dr. Jean-Pierre Van Buyten**<br>*VITAZ*

	**Dr. Bart Billet**<br>*AZ Delta*<br><br>**Dr. Sofie Denkens**<br>*Heilig Hart Ziekenhuis Lier*

4 Introduction

Burden of Persisting Spinal Pain Syndrome Type II

When surgical spine interventions fail, patients may remain with persistent pain, termed Persistent Spinal Pain Syndrome Type II (PSPS T2). The incidence of PSPS T2 after lumbar spinal surgery is estimated around 10-40%, depending on the type of surgery[1,2]. PSPS T2 is associated with chronic (low back and/or leg) pain and disability[3], which severely impacts quality of life (e.g., participation in social life and society) and mental health (e.g., high psychological morbidity)[4,5].

Burden of opioid use

PSPS T2 is categorised under high-impact chronic spinal pain, with a substantial activity restriction. Patients with PSPS T2 usually have a long-standing history of pain and are prescribed opioids that might be dosed over five times the morphine equivalent daily dose[6]. Although the use of opioids is controversial and currently recommended for only a short period of time, opioids are a valuable world-wide option in the armamentarium of clinicians treating patients with PSPS T2[7,8]. Despite recommendations and guidelines to avoid doses above 90 MME (morphine milligram equivalents)[9-11], more than 24.6%[12] up to 39.3%[13] of chronic non-cancer pain patients receives doses ≥ 90 MME. Belgium belongs to the top three European countries in defined daily dose analgesic opioids use[14]. Long-term opioid use is associated with constipation, tolerance, hyperalgesia, respiratory depression, sedation, addiction and the prominent and aversive withdrawal symptoms that manifests when opioid use is discontinued[15]. Avoidance of the opioid withdrawal syndrome is a frequently cited reason for continued opioid use[16]. Thus, a complex clinical challenge has raised because reducing opioids is likely to increase patient's pain intensity, whereas increasing or sustaining opioid doses will lead to changes in pain sensitivity and increase the risk of opioid addiction[17]. Spinal Cord Stimulation (SCS) may therefore reduce the need for long-term pain medication[18].

Treatment of PSPS T2

Treatment of PSPS T2 is complex as the condition involves neuropathic and/or nociceptive elements[19]. When conservative treatment does not achieve adequate pain relief, SCS is proposed as a minimally invasive treatment option[19]. SCS is a type of neuromodulation that involves the implantation of an epidural electrode, connected through extensions with a subcutaneous implanted pulse generator[20]. Electrical pulses at different frequencies are generated and delivered to the spinal cord[21]. The goal is to reduce chronic pain , leading to benefits in terms of functionality and health-related quality of life (QoL)[22,23]. Especially given the current opioid crisis and the common side effects of high-dose opioid analgesics, SCS has proven to be the treatment choice above opioids to alleviate chronic pain[24].

Does treatment with SCS really decrease medication use? Previous results.

The effectiveness of SCS has already been proven for reducing low back and leg pain, increasing functionality, and optimizing health-related quality of life[22-25]. Additionally, SCS is cost-effective for patients with PSPS T2[26]. Recently, a meta-analysis evaluated the effect of SCS on opioid intake and pain medication reduction in patients with intractable low back and/or leg pain[27]. The authors reported that the odds of reducing opioid consumption were significantly increased when being treated with SCS compared to conventional medical care (OR 8.60 (95% CI from 1.93 to 38.30))[27]. Nevertheless, despite a significant decrease in the amount of opioids, the number of patients that could effectively reduce or fully eliminate opioid use after SCS, was rather limited (ranging from only 7 to 41%)[27].

Opioid tapering before initiating neuromodulation: previous results

In an earlier large multicentre cohort study in Belgium that compared pain medication use before SCS and after 12 months of SCS, we noted a statistically significant decrease in pain medication use. However, the actual number of patients taking opioids did not change drastically, i.e., 73.6% at baseline versus 62.9% at 12 months[18].

In patients with intrathecal opioid therapy, Grider et al. revealed the efficacy of a pretrial elimination of systemic opioids followed by a period of abstinence[28]. In 2018, a retrospective study in 60 chronic non-cancer pain patients confirmed the success rate of this concept[29]. When evaluating patients 1 year after SCS implantation, compared to pre-implantation, patients who reduced/eliminated their opioids showed significantly lowered pain intensity scores from 7.61 ± 0.28 to 5.42 ± 0.55 (P<0.001)

at 1 year. Patients who remained on their pre-implantation opioid dosage reported only a modest decline in pain intensity (from 6.95 ± 0.34 to 5.5 ± .065 (p=0.03))[30]. In line with this reasoning, it is expected that the results of SCS will be further improved when patients are no longer taking opioids before SCS implantation and that patients can become opioid free after SCS implantation. This study aims to eliminate all use of opioids before SCS, to start the SCS trial opioid-free and improve the chances of remaining opioid free 12 months post-surgery.

This approach of first performing an opioid tapering protocol is already used in clinical practice. Clonidine is administered to minimize the symptoms of withdrawal with a fixed dose per day[31,32]. Based on a retrospective study in Belgium, the effectiveness, safety, and feasibility of this approach was demonstrated, with tolerable withdrawal symptoms for patients[33]. In parallel, a different personalized tapering protocol has been used in chronic non-cancer pain patients with opioid use [34,35], in which opioid substitution treatment is implemented with the partial μ-opioid receptor agonist buprenorphine (analgesic ability), combined with naloxone (to reduce the risk of IV medication use). This protocol[36,37] has recently been validated in the context of chronic pain with good long-term result regarding pain parameters, opioid use, opioid craving and quality of life[34,35]. Based on patient's pain scores and severity of withdrawal symptoms, exact doses are individually regulated, leading to a personalized tapering protocol[34,35].

Therefore, we propose to start with **a pain medication tapering protocol** *before starting the SCS trajectory as the* **most promising treatment strategy** *for patients implanted with SCS. This allows us* **to tackle the high burden of** *patients that take a lot of* **pain medication** *by proceeding towards a more logical treatment plan for a costly and debilitating condition.*

5 Study Schematic/Methods

5.1 Study Design

This study is a multicenter three-arm randomized controlled trial to evaluate whether pain medication tapering before SCS implantation alters disability after 12 months in PSPS T2 patients scheduled for SCS implantation compared to no pain medication tapering.

5.2 Research methodology

Patients will be recruited for this study after being scheduled for a treatment trajectory with SCS (see Figure 1, section 19). After inclusion, patients will complete the baseline assessment, assessing socio-demographic information, baseline clinical data, MINI neuropsychiatric interview and questionnaires related to our primary and secondary outcome measures. Subsequently, patients will be randomized into one of the three study arms, as presented in Figure 2, section 19. Patients allocated to one of the tapering arms will be hospitalised for six days, which can be extended to eight days, if necessary, according to the physician. The standardized tapering group will receive medication until the SCS trial period, whereas the personalized tapering group will receive medication for four to six weeks after IPG implantation. At least three weeks after hospitalization, patients receive a SCS trial followed by a definitive implantation in case the trial period was successful. Patients are seen by the investigator at one month, three months, six months, and twelve months after definitive implantation to complete the follow-up assessments according to the study protocol.

5.3 Patient recruitment

The treating neurosurgeon or anesthesiologist will be responsible for recruitment. Patients will be recruited for the study after being scheduled for a treatment trajectory with SCS. The treating neurosurgeon or anesthesiologist will inform the investigators if a patient is eligible for SCS. Afterwards, the investigator will contact the patient to perform a study-related eligibility screening and provide information about the study. Patients will be recruited from 4 centers that are already cooperating for other studies: UZ Brussel, AZ Delta, Heilig Hart Ziekenhuis Lier and Vitaz.

5.4 Baseline assessments

At baseline assessments, patients are asked about their demographics and socio-economic status (sex, age, marital status, years of education, educational level, employment status, occupation, income, and household members) and baseline clinical data (number of previous surgeries, duration of pain, how many years they have used pain medication) is collected. In addition, a retrospective questionnaire about their health care use over the period of three months prior to baseline assessment is administered. The MINI neuropsychiatric interview[38] will be administered in order to determine major psychiatric comorbidities. Further, patients will receive questionnaires related to our primary and secondary outcome measures, as described below.

5.5 Outcome measures

Primary outcome measure: disability
Disability will be assessed using the **Oswestry Disability Index (ODI)**. *The ODI evaluates functional disability due to low back pain[39,40] and contains the following topics: pain intensity, lifting, ability to care for oneself, ability to walk, ability to sit, sexual function, ability to stand, social life, sleep quality and ability to travel. Each topic is scored on a Likert scale from 0 (no disability) to 5 (maximum disability possible). The scores for all questions are summed and multiplied by two to obtain the index (range 0–100), with high scores representing high disability. In PSPS T2, the minimum clinically significant difference in ODI score is 9.0 with a sensitivity of 0.74 and a specificity of 0.92[41].*

Secondary outcome measure: pain intensity
Current pain intensity and mean pain intensity during the past 7 days will be assessed using the **Visual Analogue Scale (VAS - 100 mm)**, *separately for leg and low back pain. The VAS pain score is a reliable measure, being valid and sensitive to change[42,43]. The minimal clinically important difference in VAS score for chronic low back pain is about 18-19 mm[44]. VAS has a good test-retest reliability[45].*

Secondary outcome measure: health-related quality of life
To describe health-related quality of life, the **EuroQol with five dimensions and five levels (EQ-5D-5L)** *will be used[46]. Dimensions include mobility, self-care, usual activities, pain/discomfort, and anxiety/depression, where patients are asked to choose from five boxes with statements ranging from 'no problems' to 'extreme problems'. The EQ-5D-5L index scores range from 0 to 1, with 0 and 1 corresponding respectively to death and full health, based on preference-weighted health state classification algorithms. The EQ-5D-5L has been validated in low back pain patients[47,48] and a Belgian EQ-5D-5L value set is available[49].*

Secondary outcome measure: participation
The **Impact on Participation and Autonomy Questionnaire (IPA)** *will be used to measure participation and autonomy[50,51]. This questionnaire provides a measure of limitations in participation and autonomy and includes 39 questions across 5 domains: autonomy indoors, autonomy outdoors, family role, social life and relationships, and work and education. Participants rate each item on a Likert scale from 0 (very good) to 4 (very poor). The scoring captures how likely respondents feel they will be able to participate in a described activity and how their disability impacts their ability to participate. Higher scores indicate more important restrictions. The IPA has good psychometric properties, including reliability, validity, and responsiveness to intervention[51,52].*

Secondary outcome measure: domains affected by substance use
To evaluate domains affected by substance use, the following questionnaires will be used; the **(i) Measurements in the Addictions for Triage and Evaluation (MATE)** *is a structured interview used to assess drug (including opioid) related patient characteristics and problems[53]. It evaluates the use of psychoactive substances, history of substance use treatment, and substance craving[53]. In addition, it establishes social engagement, identifies contextual influences on engagement and the resulting need for treatment[53]. A* **(ii) VAS for opioid craving (OCVAS)** *is used to assess between 'no craving at all' and 'strongest craving ever', measuring severity of opioid craving[54]. The* **(iii) Current Opioid Misuse Measure (COMM)** *is a 17-item questionnaire that assesses the risk for aberrant medication-*

related behaviour. Each question is graded from 0 (never) to 4 (very often) resulting in a total score between 0 and 68. The questionnaire is a reliable and valid tool in patients with chronic pain who are prescribed opioids for their pain[55].

<u>Secondary outcome measure: medication use</u>
The **Medication Quantification Scale III (MQS III)** *will be used to quantify pain medication regimens in a wide variety of pain conditions[56]. It provides a numerical output that represents the negative impact of each medication[57]. For each medication, a MQS score is calculated by multiplying a detriment weight for a given pharmacologic class with a score for dosage[58]. Medication is subdivided into five classes: non-steroidal anti-inflammatory drugs (NSAIDs), muscle relaxants, neuropathic pain medications (antidepressants and anticonvulsants), benzodiazepines and opioids. All calculated values are summed to obtain a total MQS score.*

<u>Secondary outcome measure: healthcare expenditure and indirect costs</u>
Healthcare expenditure *will be investigated by two means. For the expenditures associated with in-hospital care, data will be extracted from hospital claims data. The other costs are collected through self-reporting methods, for which a combination of diaries and questionnaires will be used. Absence from work (indirect costs) is documented via those questionnaires and valued using the human capital approach. Hence, healthcare expenditure includes the number of days spent in hospital, medical tests related to post-operative surgery and any kind of post-surgical treatments (e.g., pain medication, physiotherapy).*

<u>Secondary outcome measure: psychological constructs</u>
(I) The **Hospital Anxiety and Depression Scale (HADS)** *aims to measure symptoms of anxiety and depression and consists of 14 items: seven items for the anxiety subscale (HADS Anxiety) and seven for the depression subscale (HADS Depression). Each item is scored on a response-scale with four alternatives ranging between 0 and 3. After adjusting for six items that are reversed scored, all responses are summed to obtain the two subscales. Recommended cut-off scores are 8–10 for doubtful cases and ≥ 11 for definite cases[59]. HADS was found to perform well in assessing the symptom severity of anxiety disorders and depression in both somatic, psychiatric, and primary care patients and in the general population[60].*

(II)The **General Self-Efficacy (GSE) scale** *is a 10-item self-report questionnaire and will be used to assess perceived self-efficacy. Self-efficacy is the belief in one's competence to cope with challenging demands and the belief in one's capabilities to achieve goals[61-63]. Self-efficacy beliefs determine whether coping behaviour is initiated, how much effort is expended, and how long this behaviour is sustained in the face of obstacles and aversive experiences[64]. Higher self-efficacy scores have shown to be associated with lower pain intensity levels, better physical health-related-QoL, better mental health-related-QoL, lower catastrophizing and use of coping strategies[65]. The General Self-Efficacy Scale has been used in chronic lower back pain patients and musculoskeletal pain[66,67]. Its validity was assessed in participants dealing with stressful health-related situations[62].*

(III) The **Pain Catastrophizing Scale (PCS)** *is a self-reported questionnaire used to assess catastrophic thoughts or feelings accompanying the previously experienced pain[68,69]. It consists of 13 items that evaluate 3 subscales of catastrophizing: rumination, magnification and helplessness on a 5-point Likert scale [68,69]. The PCS factor scales are valid and reliable in chronic pain[68-70].*

(IV) The **Multidimensional Pain Inventory (MPI)***, a 61-item questionnaire, will be used to assess the coping strategy profile. This profile is made up out of the behavioral and psychological response to chronic pain experience. The MPI is divided in three parts. Part I evaluates five dimensions of the pain experience. Part II examines the responses of significant others to communications of the patients' pain. Part III assesses the patients' report of their participation in common daily activities. The MPI is valid in chronic pain patients with indications for SCS implant[71].*

Secondary outcome measure: sleep quality

The **Pittsburgh Sleep Quality Index (PSQI)** *is a self-rated questionnaire used to measure sleep quality over the prior month[72,73]. It includes 24 items and 7 component scores: subjective sleep quality, sleep latency, sleep duration, habitual sleep efficiency, sleep disturbances, use of sleeping medication, and daytime dysfunction[73]. It is a reliable and valid measurement tool with good internal consistency and construct validity in chronic pain patients[74].*

Secondary outcome: central sensitization

The **Central Sensitization Inventory (CSI)** *will be used to assess symptoms associated with central sensitization[75]. The total score of the CSI ranges between 0 -100. The CSI has good psychometric properties including initial construct validity, test-retest reliability, good discriminative power, and good internal consistency in patients with chronic pain[76].*

5.6 Early study termination

In case of a negative SCS-trial, patients will be excluded from the study but already collected data will be preserved for analysis.

5.7 End of study

The end of study for an individual patient is reached when the patient has completed all study procedures, including the 12-month follow-up assessment, as described in this protocol (see section '10. Study Assessments and Procedures'). Patients can indicate in the Informed Consent Form whether the investigators may contact them until six months after study end, for process evaluation purposes.

Overall, the end of the study is reached when the last follow-up assessment of the last patient in the last participating site is completed.

As soon as the entire study has ended, the sponsor will be notified, so that the Competent Authority and Ethics Committee can be informed in a timely manner according to the regulatory requirements (within 90 days after end of the study, or if the study terminates early, this period must be reduced to 15 days and the reasons should clearly be explained).

5.8 Sample size calculation

The sample size calculation was performed on the primary outcome measure, i.e., "disability, measured with the self-reporting questionnaire ODI", at 12 months. The following assumptions were made:

- *Based upon earlier data[77], the mean disability score at 12 months with SCS was 33.34 (SD: 16.86).*
- *The expected difference between tapering and no tapering at 12 months of SCS is 19.64%[30], leading to an expected total ODI score of 26.79 in the tapering groups.*
- *A 10% decrease in ODI score is assumed with the personalized tapering protocol compared to a fixed tapering protocol (assumption since no ODI data is available to estimate the actual beneficial effect).*
- *The common standard deviation (16.789) is based upon 4 repeated measures from an earlier trial in an identical study population[77].*
- *The sample size calculation was performed for achieving an 80% power at the 5% level of significance with Bonferroni correction for multiple comparisons. An equal allocation ratio was assumed for each study arm.*

To conduct a three-arm parallel group, randomised clinical trial with 5 assessments, and estimated true mean ODI responses for the no tapering group of 33.34, fixed tapering group of 26.79 and personalized tapering group of 24.11, with a common standard deviation of 16.789, and 80% power to detect differences in means at a 5% two-sided significance level with Bonferroni correction, a total

sample size of 147 patients is needed. This calculation was performed by the guidelines provided in the book of Chow et al. concerning sample size calculations in clinical research[78] *and controlled with G power version 3.1. To account for a SCS trial failure rate of 11.3% according to Belgian reimbursement rules*[77] *and a general loss-to-follow-up of 20%, a sample size of 65 patients per arm will be needed. This results in a total sample size of 195 patients for this study.*

6 Study Objectives and Endpoints

6.1 Primary Objective

The primary objective of the study is to examine whether there is a difference in disability after 12 months of SCS in PSPS T2 patients after receiving a standardized pain medication tapering protocol before SCS implantation, a personalized pain medication tapering protocol before SCS implantation, or no pain medication tapering protocol before SCS implantation.

6.2 Secondary Objectives

The secondary objective of the study is to examine whether there is a difference after 12 months of SCS in PSPS T2 patients after receiving a standardized pain medication tapering protocol before SCS implantation, a personalized pain medication tapering protocol before SCS implantation, or no tapering protocol before SCS implantation on pain intensity, health-related quality of life, participation, domains affected by substance use, anxiety and depression, medication use, psychological constructs, sleep, central sensitization, and healthcare expenditure.

6.3 Endpoints

The **primary** *endpoint is to observe whether there is a difference in the* **ODI** *score in PSPS T2 patients* **12 months after definitive SCS implantation** *between the three arms. A longitudinal mixed model analysis will be used with timepoints defined at baseline, 1 month, 3 months, 6 months, and 12 months after definitive SCS implantation.*

The **secondary** *endpoint is whether the three arms differ on the VAS, EQ-5D-5L, IPA, MATE, HADS, OCVAS, COMM, MQS III, GSE, PCS, MPI, PSQI, CSI and Health Expenditure after definitive SCS implantation. Longitudinal mixed model analysis will be used with timepoints defined at baseline, 1 month, 3 months, 6 months, and 12 months after definitive SCS implantation.*

7 Selection of Patients

7.1 Inclusion Criteria

Patients being diagnosed with PSPS T2, defined as patients suffering from neuropathic pain of radicular origin with pain in the lower back and/or leg(s), of an intensity of at least 4/10 on the Numeric Rating Scale, for a period of at least 6 months after a minimum of one anatomically successful spinal surgery and being refractory to conservative treatment (according to Belgian reimbursement rules from January 1st, 2018). Patients need to be scheduled for SCS to be eligible for participation in the study. Moreover, patients must be 18 years or older, taking opioids, and be able to speak and read Dutch or French.

7.2 Exclusion Criteria

Exclusion criteria include the following:

- *Being actively treated for cancer,*
- *Having a life expectancy below 6 months,*
- *Receiving intrathecal drug delivery,*

- *Having contraindications for Clonidine (e.g., known hypotension which requires medication) or for Buprenorphine/Naloxone (e.g., severe respiratory insufficiency, hepatic insufficiency)*
- *Having epilepsy currently treated by Pregabalin,*
- *Currently using benzodiazepines at doses more than 40 mg diazepam-equivalents per day.*

8 Screening and Randomization

8.1 Screening and Enrollment

Patients will be informed by their treating neurosurgeon or anesthesiologist about the study. If the patient is interested, the physician will provide the patient's contact details to the study team. An investigator will then contact the patient by telephone in which the patient will be screened based on in- and exclusion criteria.

8.2 Randomization

Randomization occurs by computer-generated random number sequence. Stratified randomization will be used according to the Medication Quantification Scale III score within each centre. A 1:1:1 allocation will be used. An independent researcher will have direct access to the randomization list (one list for all centres). This independent researcher will arrange practical issues such as appointment times with the hospital wards to perform the experimental interventions.

8.3 Blinding Procedures

The statistician and outcome assessors will be blinded to group allocation. Patients cannot be blinded as a hospital stay of at least six days is necessary in both the intervention arms.

9 Interventions/Treatment

9.1 Treatments Administered

9.1.1 Control intervention: no tapering

When randomized to the control group, patients (N = 65) will not undergo a tapering program before SCS implantation. Patients first undergo a SCS trial period, followed by Implantable Pulse Generator (IPG) implantation in case of a successful trial period (50% pain reduction and 50% reduction in pain medication use, according to the current Belgium reimbursement rules). After implantation, patients are seen by the treating physician, pain nurse(s) and/or delegates of the companies of the SCS devices to program the SCS parameters. On top of that, they have a fixed 6-month follow-up appointment to re-evaluate the therapy and evaluate medication use in combination with SCS. Each hospital can continue usual care as normally provided to patients after SCS implantation. However, their healthcare expenditure will be documented using a weekly self-report.

9.1.2 Experimental Intervention: standardized tapering protocol

For each patient within the standardized tapering group (N=65), a standardized protocol will be delivered to wean off opioids and gabapentinoids before neuromodulation. Patients are to be hospitalised for six days. This might be extended to eight days, if deemed necessary, according to the physician. A schematic overview of this tapering protocol is presented in Figure 3 (section 19).

<u>During hospital stay for tapering</u>
At admission, all opioids and gabapentinoids (if taken) will be stopped, and clonidine diluted in saline (1200µg/48mL) will be administered to counteract withdrawal symptoms. Over the following hospital days, clonidine will be decreased with 150µg per day until the end of hospitalization. Throughout

the protocol, auxiliary medication including pain medication, is provided as needed in terms of symptom control. Clonidine is a centrally acting antihypertensive drug. Therefore, blood pressure and heart rate will be monitored during the hospital stay. The drip of clonidine will be temporarily lowered to a rate of 1mL/h in case of a heart rate between 40 and 45 bpm. In case of symptomatic hypotension or a heart rate lower than 40 bpm, clonidine will be stopped. In the case of symptomatic hypotension without bradycardia, a fluid challenge bolus of 250 mL will be administered, with the possibility of an additional 250 mL of saline. This protocol has been implemented and is published by the principal investigator[33].

Coaching
During the hospital admission, patients will receive videos on a daily basis from the researchers with the aim to increase motivation and persistence during the tapering, and to offer tools to prevent relapse and to cope with pain after the hospital stay.

After hospital stay for tapering
After the hospital stay, all patients will continue to use clonidine (75μg per day) up to the SCS-trial.

9.1.3 Experimental Intervention: personalized tapering protocol

Patients randomized to the personalized tapering protocol (N=65), will be switched to the partial opioid agonist Buprenorphine/Naloxone before neuromodulation. Patients will be hospitalised for six days to induce them on Buprenorphine/Naloxone (BuNa). The hospital stay might be extended to eight days, if deemed necessary according to the physician. As of hospital discharge, patients will remain on a stable BuNa dose for four to six weeks after definitive SCS implantation, after which BuNa will be tapered off. A schematic overview of this tapering protocol is presented in Figure 4 (section 19).

Before the hospital stay for tapering
If necessary, patients will start with a substitution of long-acting opioids to short-acting oxycodone at home. Per patient, a medication scheme will be provided, based on their medication use. For patients who only use short-acting opioids, no substitution is needed prior to hospital admission.

During the hospital stay for tapering
Patients will be hospitalised for six days. During the first two days, if necessary, substitution to oxycodone will be initiated and assessed, or continued when patients started the substitution at home. On the morning of the third hospital day, oxycodone is stopped and BuNa is initiated as of the appearance of withdrawal symptoms (as described by Ellerbroek et al[79]). For BuNa induction purposes, pain, craving and withdrawal are evaluated at least 4 times per day using the Subjective Opioid Scale (SOS) and the Clinical Opiate Withdrawal Scale (COWS)[80]. VAS pain and VAS craving will also be measured. Upon withdrawal symptoms (assessed as a score >13 on COWS and corroborated by positive SOS-score) BuNa is provided at a starting dose of 4/1mg BuNa. Upon withdrawal symptoms, BuNa dose will be increased by increments of 2/0.5mg as needed and until a maximum dose of 24/6mg on the first day of BuNa induction. On the morning of day 4 and 5 of the hospital admission, patients receive the entire dosage of BuNa from the previous day, and withdrawal symptoms are further assessed (identical to day one). If needed additional BuNa is given by increments of 2/0.5mg with a maximum second-day dose of 36/9mg. On the morning of day 6, patients receive the entire dosage of BuNa from the previous day. On this final day of hospitalization, a stable dose is evaluated, and minor changes are possible (minor dose reduction, minor dose increases, or dose breaking up to three times daily). Dosing will be increased if the patient experiences withdrawal and decreased if the patient experiences drowsiness. If the hospital stay is prolonged to eight days, BuNa will be provided similar to days four, five and six until a stable dose is reached. Throughout the protocol, auxiliary medication including pain medication, is provided as needed in terms of symptom control. This protocol has been used in non-cancer chronic pain patients with good results on stability of use, pain relief and craving[35].

Coaching

During the hospital admission, patients will receive videos on a daily basis from the researchers with the aim to increase motivation and persistence during the tapering, and to offer tools to prevent relapse and to cope with pain after the hospital stay.

After the hospital stay for tapering

After the hospital stay, patients remain on a fixed dose of BuNa until 4-6 weeks after the definitive SCS implantation, followed by the tapering of BuNa, as shown in Figure 4 (section 19). Whether patients remain at fixed BuNa dosage for four or six weeks depends on the initial daily dosage. Lower doses will be able to be tapered more rapidly compared to higher doses (see Figure 4).

Gabapentinoids (if taken) will also be tapered after the definitive SCS implantation. Gabapentin and Pregabaline will be tapered at a rate of 50% per week. Tapering will therefore take two weeks.

9.2 Summary of Product Characteristics

The Summary of Product Characteristics (SmPC) of Clonidine, short-acting Oxycodone and BuNa are added in Annex. The SmPC provides information on the qualitative and quantitative composition, pharmaceutical form, clinical particulars and pharmacological properties and particulars.

9.3 Direction of administration and dosing regimen

Clonidine

During the hospital stay for tapering, clonidine will be administered intravenously with a rate of 2mL/h to reach a maximum dose of 1200µg/48mL. This maximum dose will be decreased with 150µg a day until the end of the hospitalization after six (or maximum eight) days. After the hospital stay for tapering, oral clonidine (75µg per day) will be prescribed up to the SCS trial.

Short-acting Oxycodone

Oxycodone will be administered orally. The dose of oxycodone is calculated based on the patient's current opioid use. The conversion is done according to the opioid conversion tables. The converted dose will already be reduced by 25%. The maximum dose thus depends on the daily dose taken by the patient but will only be 75% of their current opioid use.

Buprenorphine/Naloxone

BuNa is administered sublingually. Patients start with a dose of 4/1mg BuNa. As of the appearance of withdrawal symptoms, BuNa can be up titrated up to a maximum daily dose is 36/9mg on the final day, based on withdrawal symptoms. The patient will remain on this scheme until 4-6 weeks after definitive SCS implantation (as described prior). Four to six weeks after definitive SCS implantation BuNa will be tapered, as presented in Figure 4 (section 19).

9.4 Storage, Labeling and Delivery

The hospital pharmacy will be responsible for storage, labeling and delivery Clonidine, short-acting Oxycodone and BuNa.

9.5 Study Disposal and Destruction

The residual study medication will be collected at the follow-up assessment at 3 months, as part of the quality assessment (compliance to the study protocol). The medication will then be given to the pharmacy of the Universitair Ziekenhuis Brussel for destruction.

10 Study Assessments and Procedures

Table 1 provides a schematic overview of the study assessments.

Table 1: Study schematic; (P) = only applicable in personalized tapering protocol; x = only question one of the MATE will be assessed*
Abbreviations: SCS = Spinal Cord Stimulation; SOC = standard of care; M = month; ODI = Oswestry Disability Index; VAS = Visual Analogue Scale; EQ5D5L = Euro Quality of Life with five dimensions and five levels; IPA = Impact on Participation and Autonomy Questionnaire; MATE = Measurements in the Addictions for Triage and Evaluation; HADS = Hospital Anxiety and Depression Scale; OCVAS = Opioid Craving Visual Analogue Scale; COMM = Current Opioid Misuse Measure; MQS III = Medication Quantification Scale III; GSE = General Self-Efficacy Scale; PCS = Pain Catastrophizing Scale; MPI = Multidimensional Pain Inventory; PSQI = Pittsburg Sleep Quality Index; CSI = Central Sensitization Inventory; HE = Health Expenditure; Q = questionnaire; COWS = Clinical Opiate Withdrawal Scale; SOS = Subjective Opioid Scale

STUDY PERIOD

		SCREENING	BASELINE	TAPERING: HOSPITAL STAY	TAPERING: HOSPITAL DISCHARGE	SCS-TRIAL (SOC)	DEFINITIVE SCS IMPLANT (SOC)	1M	3M	6M	12M
IN- AND EXCLUSION CRITERIA		x									
INFORMED CONSENT			x								
DEMOGRAPHICS AND SOCIO-ECONOMIC STATUS			x								
BASELINE CLINICAL DATA			x								
MINI NEUROPSYCHIATRIC INTERVIEW			x								
OUTCOME MEASURES	ODI		x					x	x	x	x
	VAS-PAIN		x	(P)	x	x	x	x	x	x	x
	EQ5D5L		x					x	x	x	x
	IPA		x					x	x	x	x
	MATE		x					x*	x*	x*	x*
	HADS		x					x	x	x	x
	OCVAS		x	(P)	x	x	x	x	x	x	x
	COMM		x								x
	MQS III		x					x	x	x	x
	GSE		x					x	x	x	x
	PCS		x					x	x	x	x
	MPI		x					x	x	x	x
	PSQI		x					x	x	x	x
	CSI		x					x	x	x	x
	HE-RETROSPECTIVE Q		x						x	x	x
	HE-WEEKLY SELF-REPORT		x		x	x	x				
COWS AND SOS				(P)							
URINE SAMPLE						x		x	x	x	x

10.1 Screening

The screening of the eligibility criteria for the study will be performed by the investigator during a telephone contact with the patient. This will happen after the investigator receives information whether the patient might be eligible for the study by the neurosurgeon or anesthesiologist.

10.2 Baseline

After obtaining informed consent, baseline assessments (as described in section 5.4) are administered. The baseline assessment is scheduled six to eight weeks prior to the SCS-trial.

10.3 Treatment period: hospital stay

In the personalized tapering, pain, craving, and withdrawal symptoms are measured at least 4 times per day using the VAS-pain, the VAS for opioid craving and the Subjective Opioid Scale and the Clinical Opiate Withdrawal Scale, respectively. At hospital discharge, the VAS-pain and the VAS for opioid craving will be assessed in both tapering groups. The hospital stay is scheduled three weeks prior to the SCS-trial.

10.4 SCS trial and definitive SCS implantation

Both the SCS-trial and definitive SCS implantation are standard-of-care. However, for study purposes at both moments the VAS-pain and the VAS for opioid craving will be assessed. Additionally, at admission for SCS trial implantation, a urine sample is collected.

10.5 Follow-up assessments

The follow-up visits will take place **1 month, 3 months, 6 months, and 12 months** *after definitive SCS implantation. At each follow-up visit, patients will complete the outcome measurements as indicated in Table 1, and a urine sample will be collected. The last two follow-up visits will be scheduled together with the mandatory follow-up visits every 6 months in relation to national reimbursement rules, to limit lost-to follow-up.*

10.6 Assessment Types

Healthcare expenditure will be assessed by weekly self-reports in paper format and self-reported questionnaires via Qualtrics. All interviews will be conducted by the investigator or the psychologist for the psychological assessments.

At fixed time points (at admission for the SCS-trial implantation, at 1 month, 3 months, 6 months and 12 months follow-up) a urine sample is collected. This sample will be analyzed (by urine dipsticks for opioids, benzodiazepines, gabapentin and pregabalin) at the time of collection and will be destroyed immediately after.

11 Safety Monitoring and Reporting

11.1 Adverse Events and Adverse Reactions

11.1.1 Definitions and Reporting

An adverse event is defined as an unexpected medical occurrence in a participant after exposure to a medicine or treatment, which is not necessarily caused by that medicine or treatment. Adverse

reactions are defined as all untoward and unintended responses to medicinal product related to any dose administered.

11.1.2 Reporting Period

In both opioid tapering groups, the percentage of patients that reports an AE possibly related to the treatment and the total number of AEs reported will be systematically recorded from baseline assessment until 12 months after definitive SCS implantation. Different intermediate follow-up assessments will be organized to closely monitor treatment effects (at 1, 3, 6, and 12 months after definitive SCS implantation). The cooperating centers will report all AEs to the sponsor.

11.1.3 Intensity

During the study, the investigator will grade the intensity of any adverse event or reaction as follow:

Mild: Symptoms do alter patient's normal functioning.
Moderate: Symptoms produce some degree of impairment to function, but are hazardous, uncomfortable, or embarrassing to the patient.
Severe: Symptoms hazardous to well-being, significant impairment of function or incapacitation.

11.1.4 Relationship to treatment

The steering committee will determine the relationship to the treatment:

Definitely related: There is clear evidence to suggest a causal relationship, and other possible contributing factors can be ruled out. The clinical event, including an abnormal laboratory test result, occurs in a plausible time relationship to the study intervention and cannot be explained by concurrent disease or other drugs or chemicals.
Probably related: There is evidence to suggest a causal relationship, and the influence of other factors is unlikely. The clinical event, including an abnormal laboratory test result, occurs within a reasonable time after administration of the study intervention, is unlikely to be attributed to concurrent disease or other drugs or chemicals.
Potentially related: There is some evidence to suggest a causal relationship (e.g., the event occurred within a reasonable time after administration of the study intervention). However, other factors may have contributed to the event (e.g., the participant's clinical condition, other concomitant events). Although an AE may rate only as "possibly related" soon after discovery, it can be flagged as requiring more information and later be upgraded to "probably related" or "definitely related", as appropriate.
Unlikely to be related: A clinical event, including an abnormal laboratory test result, whose temporal relationship to the study intervention makes a causal relationship improbable (e.g., the event did not occur within a reasonable time after administration of the study intervention) and in which other drugs or chemicals or underlying disease provides plausible explanations (e.g., the participant's clinical condition, other concomitant treatments).
Not related: The AE is completely independent of the study intervention, and/or evidence exists that the event is definitely related to another etiology. There must be an alternative, definitive etiology documented by the clinician.

11.2 Serious Adverse Event or Reaction

11.2.1 Definitions

A serious adverse event or serious adverse reaction is defined as any untoward medical occurrence or response to a medicinal product or treatment that at any dose:

- *Results in death*
- *Is life-threatening*
- *Results in patient hospitalization*
- *Results in a persistent or significant disability/incapacity*

- *Results in congenital anomalies/birth defects*
- *Important adverse events that are not immediately life-threatening or do not result in death or hospitalization but may jeopardize the patient or may require intervention to prevent one of the outcomes listed.*

A life-threatening adverse event is any adverse drug experience that places the patient in immediate risk of death from the reaction as it occurred, i.e., it does not include a reaction that, had it occurred in a more severe form, might have caused death.

11.2.2 Immediate Reporting

In both treatment groups, the percentage of patients that is reporting an SAE possibly related to the treatment and the total number of SAEs reported will be systematically recorded from baseline assessment until 12 months after definitive SCS implantation. The cooperating centers will report all SAEs to the sponsor within 24 hours of becoming aware. All SAEs will be reported to the Competent Authorities and Ethics Committee within 24 hours.

11.3 Suspected Unexpected Serious Adverse Reaction (SUSAR)

11.3.1 Definitions

A SUSAR is defined as a serious adverse reaction that occurs in a clinical trial participant, which is assessed as being suspected as well as unexpected, and which is having a reasonable possibility of having a causal relationship with the study medication.

11.3.2 Reporting

All SUSARs must be reported to the ethics committee and health authority as per EU and Belgian legislation. The reporting period for SUSARs if fatal or life-threatening is as soon but not later than 7 days after becoming aware. For SUSARs not fatal or non-life threatening the reporting period is 15 days after becoming aware. The sponsor will report to the Ethics Committee and Health Authorities.

11.4 Other safety data requiring an immediate declaration

In accordance with the applicable regulatory requirements, the investigator will notify the Ethics Committee and Health Authorities of any suspected unexpected serious adverse reactions.

11.5 Procedures for Handling Special Situations

11.5.1 Overdose Management

Clonidine

Symptoms:
Manifestations of intoxication are due to a generalized sympathetic depression and include pupillary constriction, lethargy, bradycardia, hypotension, hypothermia, somnolence including coma and respiratory depression including apnea. Paradoxical hypertension caused by stimulation of peripheral alpha1- receptors may occur. Transient hypertension may be seen if the total dose is over 10 mg.
Management:
There is no specific antidote for clonidine overdose. Administration of activated charcoal should be performed where appropriate. Supportive care may include atropine sulfate for symptomatic bradycardia, and intravenous fluids and/or inotropic sympathomimetic agents for hypotension. Severe persistent hypertension may require correction with alpha-adrenoceptor blocking drugs. Naloxone may be a useful adjunct for the management of clonidine-induced respiratory depression.

Short-acting oxycodone

Symptoms:
Acute overdose with oxycodone can be manifested by respiratory depression, somnolence, progressing to stupor or coma, hypotonia, miosis, bradycardia, hypotension, pulmonary oedema, and death.

Management:
A patent airway must be maintained. The pure opioid antagonists such as naloxone are specific antidotes against symptoms from opioid overdose. Supportive measures (artificial respiration, oxygen supply, administration of vasopressors and infusion therapy) should, if necessary, be applied in the treatment of accompanying circulatory shock. Upon cardiac arrest or cardiac arrhythmias cardiac massage or defibrillation may be indicated. If necessary, assisted ventilation as well as maintenance of water and electrolyte balance. Other supportive measures should be employed as needed.

In the case of massive overdose, administer naloxone 0.8 mg intravenously. Repeat at 2-3-minute intervals as necessary, or by an infusion of 2 mg in 500 ml of normal saline or 5% dextrose (0.004 mg/ml).

The infusion should be run at a rate related to the previous bolus doses administered and should be in accordance with the patient's response. However, because the duration of action of naloxone is relatively short, the patient must be carefully monitored until spontaneous respiration is reliably re-established.

For less severe overdose, administer naloxone 0.2 mg intravenously followed by increments of 0.1 mg every 2 minutes if required.

Naloxone should not be administered in the absence of clinically significant respiratory or circulatory depression secondary to oxycodone overdose. Naloxone should be administered cautiously to persons who are known, or suspected, to be physically dependent on oxycodone. In such cases, an abrupt or complete reversal of opioid effects may precipitate pain and an acute withdrawal syndrome.

Gastric contents may need to be emptied as this can be useful in removing unabsorbed drug.

However, overdosing of oxycodone is highly unlikely given that the prescribed dose of oxycodone is only 75% of the equivalents of opioids that the patient is already taking.

Buprenorphine/Naloxone

Symptoms:
Respiratory depression as a result of central nervous system depression is the primary symptom requiring intervention in the case of overdose because it may lead to respiratory arrest and death. Signs of overdose may also include somnolence, amblyopia, miosis, hypotension, nausea, vomiting and/or speech disorders.

Management:
General supportive measures should be instituted, including close monitoring of respiratory and cardiac status of the patient. Symptomatic treatment of respiratory depression, and standard intensive care measures, should be implemented. A patent airway and assisted or controlled ventilation must be assured. The patient should be transferred to an environment within which full resuscitation facilities are available.

If the patient vomits, care must be taken to prevent aspiration of the vomitus.

Use of an opioid antagonist (i.e., naloxone) is recommended, despite the modest effect it may have in reversing the respiratory symptoms of buprenorphine compared with its effects on full agonist opioid agents.

If naloxone is used, the long duration of action of buprenorphine should be taken into consideration when determining the length of treatment and medical surveillance needed to reverse the effects of an overdose. Naloxone can be cleared more rapidly than buprenorphine, allowing for a return of

previously controlled buprenorphine overdose symptoms, so a continuing infusion may be necessary. If infusion is not possible, repeated dosing with naloxone may be required. Ongoing intravenous infusion rates should be titrated to patient response.

11.6 Annual Safety Report

An annual safety report will be provided by the sponsor.

12 Data Collection and Management

The total amount of collected data will be less than 1TB and will consist of the informed consent forms (paper format), information on patient demographics and socio-economic status (.csv files),the MINI neuropsychiatric questionnaire (paper format), questionnaire results, MINI neuropsychiatric questionnaire, ODI, VAS-pain, EQ-5D5L, IPA, MATE, HADS, OCVAS, COMM, MQS III, GSE, PCS, MPI, PSQI and CSI (.csv files), information concerning adverse events (paper format), information collected by the weekly self-reports and questionnaires for healthcare expenditure (paper format and .csv files), personal data concerning health status (.csv files) and self-written codes in R and SAS to analyze data (scripts in R and SAS readable formats).

During the study, all investigators involved will be responsible for data management and storage. Data will be stored on the VUB SharePoint (system-encrypted) with a back-up on a secure external HDD. Access permissions will be managed in SharePoint by the PI and is limited to the investigators and supervisors. After the project, the archiving location will be in the VUB University Archive for restricted access data (pseudonymized data), which will be the responsibility of the ZAP-supervisor Prof. Dr. Maarten Moens. Data will be preserved there for 25 years after the end of the study. All possible personal identifiable data (vide infra) will be removed from the archived data. Access restrictions will be applied and will be specified in a data use agreement containing following an evaluation of the re-use request by ethical committee, a non-disclosure agreement and warranties for safe storage of data.

The full dataset (including confidential data and the key) will be archived for 25 years, no deviations will be made. Data will be pseudonymized. The name of the patient will be replaced by a unique number. As an additional security measure, the file linking the pseudonymization to the original direct identifiers will be encrypted before it is uploaded on SharePoint (key).

Additional security measures concerning the research data will be made. Firstly, a written informed consent will be obtained from each patient. The informed consents will be safely stored in a closed room. Secondly, all data (including confidential data) will be stored in a system-encrypted storage location (VUB SharePoint) with restricted access, limited to the investigators, study coordinator and supervisors who are working on this project. Thirdly, a clinical trial master file will be recorded with all documentation of the study, available for internal/external reviewing. Finally, all researchers involved in this project should have a valid Good Clinical Practice certificate.

Data will be collected via web-based self-reported questionnaires that are provided to the participants by a link to the questionnaires. Collected data will include answers to validated questionnaires related to the participants' disability, pain intensity, health-related quality of life, participation, domains affected by substance use, anxiety and depression, medication use, psychological constructs, sleep, and central sensitization. In addition, participants will be asked about the number of previous surgeries, the duration of how long they are experiencing pain, how many years they have used pain medication and general socio-demographic data (e.g., sex, age, marital status, years of education, educational level, employment status, occupation, income, and household members). Further, the patients' name, telephone number, e-mail, hospital claims data and information on the type of IPG will be collected. The objective for the data collection is scientific research, as described in this study protocol. Written informed consent of the participants will be collected and provide the basis for the legal ground for the data management.

Personal data will be processed in accordance with the European Union's Data Protection Directive (Directive 95/46/EC) and regulation EC45/2001, the relevant Belgian legislation concerning data protection of July 30th 2018, and good clinical practice. As we collect personal identifiable data, following steps are taken to limit unauthorized access. Informed consents will be preserved at a secure location at the VUB. Qualtrics (Qualtrics, Provo, UT) will be used for data collection to improve data protection as responses to questionnaires will only be accessible to the investigator. Collected data will be password protected. Personal identifiable and clinical trial data will be separated, with the latter receiving a unique participant ID. Access to informed consents, personal identifiable data and the link with the participant ID will be restricted to the investigators and supervisors and stored separately from the trial data. Eventual further dissemination of data will only occur in a pseudonymized or aggregated way.

Pseudonymization will be performed as soon as data collection starts. As an additional safety measure, the file that is linking the pseudonymization with the original direct identifiers will be encrypted before uploading it on SharePoint.

13 Quality management

The medical team at each cooperating hospital will be trained in performing the tapering programs by one of members of the PIANISSIMO consortium with expertise in pain medication tapering. Once the trial is initiated, the medical teams will be able to contact the PIANISSIMO consortium directly, allowing them to discuss any difficulties experienced on short notice.

14 Statistical Considerations and Data Analysis

14.1.1 Baseline analysis

Baseline data will provide cross-sectional results on demographics, socio-economics, baseline clinical data, disability, pain intensity, health-related quality of life, participation, domains affected by substance use, anxiety and depression, medication use, psychological constructs, sleep, central sensitization, and questionnaires about healthcare expenditure for the complete PSPS T2 group and comparisons between possible subgroups. All baseline data that is collected will be included in the analysis. Furthermore, correlation analyses will be performed to unravel correlations between the different outcome measures in patients with PSPS T2, eligible for SCS. Based on the distribution of the data, subgroup differences at baseline will be assessed using a parametric test or its non-parametric alternative at alpha lower than 0.05. Correlation analysis will be performed with Pearson if the assumption of a linear relation between two variables is met, otherwise a Spearman correlation coefficient will be calculated and tested at alpha < 0.05.

14.1.2 Main analysis

Longitudinal mixed model analysis will be used to evaluate and compare therapy effects. The need for random intercepts and slopes will be evaluated. Potential confounding variables (e.g., baseline medication use) will be considered in the analysis. Statistical, as well as clinically significant differences will be defined at alpha < 0.05 and the effect size will be determined. Furthermore, based on the baseline data, we will determine predictive factors and which subgroup of patients will benefit the most of a tapering program before initiating SCS, by several machine learning techniques (both supervised and unsupervised techniques). For the prediction analysis, the model will be built on an 80% training-set and tested on a 20% validation-set. All analysis will be performed in SAS or R.

14.1.3 Health economic analysis

First, a within trial economic evaluation will be conducted[84]. All costs of all participants will be considered, starting from three months before baseline assessment until the end of the 12-month follow-up period. The health economic analysis is carried out with the intention to treat population. Data collection on the use of resources will be carried out via retrospective questionnaires and weekly self-reported diaries on healthcare use. Intervention costs will be based on the hospital claims data from the tapering admissions and the study notes documenting the duration of each stay per patient. The valuation of resource use is based on national tariffs. A societal perspective is adopted as indirect costs of productivity loss are a crucial part in the analyses. Health outcomes will be expressed in two ways. Effects are expressed in percentage disability, which is the primary outcome in this trial. Next, health outcomes will be considered, expressed in utility using health state values from the general public, in accordance to the Belgian guidelines[85]. The comparator is the control group receiving no opioid tapering. Missing data are inevitable in health economic evaluations alongside a clinical trial and will be addressed prior to economic analysis[84]. Differences in cost between both groups will be analysed using generalised linear models. Modified Park test will be used to identify the appropriate link function[86]. The overall result is expressed in an incremental cost effectiveness ratio (ICER, i.e., incremental cost divided by the percentage increment in functioning and incremental cost divided by the incremental quality-adjusted life year gained). The point estimates of incremental costs and increment health benefits (deterministic analyses) are subject to uncertainty which will be addressed in probabilistic analyses[86]. We will apply nonparametric bootstrapping to test for statistical differences in costs and health benefits to investigate the uncertainty around these outcomes and summarized in cost-effectiveness acceptability curves indicating the likelihood of the intervention to be cost-effective at any willingness-to-pay threshold. Reporting on the results of the health economic evaluation will be in line with the CHEERS-guidelines[87]. Besides the within-trial health economic evaluation, a model-based evaluation will be conducted to estimate the expected costs and health outcomes in both the control and intervention group beyond the follow-up period of the trial. A Markov-model will be developed compliant to the commonly used guidelines. We assume a cycle of 1 year in the model and apply a lifetime horizon. Lifetime incremental costs and quality-adjusted life years will be the input for the ICER calculation. Discount rates of 3% for costs and 1.5% for utilities will be applied, which is in line with the Belgian guidelines[88]. The subsequent probabilistic analyses and reporting strategies are identical to those described above.

14.1.4 Process evaluation

A process evaluation will be conducted to supplement the primary trial results (by providing contextual information to the trial results and increasing the acceptability of the trial) and analyze secondary factors that made the tapering programs successful or unsuccessful[89]. The process evaluation is rolled out based on frameworks described in literature[90,91]. Conducting a process evaluation will result in a deeper understanding of the range of effects, and provide context on how they vary among recipients of the intervention[90]. The process evaluation will allow to evaluate which types of individuals are participating in the trial and how they respond to the tapering protocols. By collection of contextual data, it is possible to take personal patient profiles into account.

Data collection for the process evaluation mainly consists of already collected data on the primary and secondary outcome measures. Additional data on patients and health care providers will be collected by organizing two focus groups. The focus groups will be organized with the purpose to collect information on the burdens, facilitators, and overall experience of the trial for both the patients and the staff members that were involved in treatment of patients during the tapering hospitalization. The focus groups are optional for both the patients and professionals. Additionally, the reason for non-participation or dropout will be inventoried in case patients provided these reasons. In the Informed Consent Form, participants can indicate whether the investigators may contact them until six months after early study termination, for process evaluation purposes.

A new submission to the Ethics committee will be made for the professionals focus group. Invitations for the professionals focus group will be sent during the hospitalization of the patients. Participation

in the professionals focus group is independent of the current trial, there will be a separate application where informed consent will be collected.

15 Ethical Considerations

15.1 Ethical conduct of the study

Approval for the conduct of the trial will be obtained from The Regulatory Authorities and the Ethics Committee of the Universitair Ziekenhuis Brussel and of each participating centre. The trial will be conducted according to the current version of the principles laid down in the Declaration of Helsinki, the European Union Clinical Trial Directive 2001/20/EC, the "Note for guidance on Good Clinical Practice" (CPMP/ICH/135/95 of 17 January 1997), and the current version of the GCP-regulation.

All diagnostic procedures and treatments applied are part of standard management of acute neuromodulation patients and will follow European and national guidelines. These procedures are therefore of immediate benefit to the patients. The investigators will assure that each patient enrolled in the trial will receive best practice medical treatment.

An annual progress report will be submitted by the sponsor to the central EC within 30 days of the anniversary date on which the favourable opinion was given, and annually until the trial is declared ended. It is the responsibility to produce the annual reports as required. The Principal Investigator will pass this annual report to all local investigators who are responsible for submission to the local EC as applicable.

Within one year after the end of the study, the Investigator will submit a final clinical study report with the results, including any publications/abstracts, to the Sponsor, which in turn submits it to the central EC. The Principal Investigator will notify the sponsor of the end of the study and the sponsor will notify the central EC of the end of the study. If the study is ended prematurely, the Investigator will notify the central EC, including the reasons for the premature termination. All correspondence with the ECs will be retained in the Trial Master File/Investigator Site File.

15.2 Informed Consent

Eligible patients will only be included in the study after providing a written informed consent. Informed consent will be obtained prior to conducting any study-specific procedures (as described in this protocol).

Before enrolling in the study, the investigator will explain the study and the implications of participation to potential participants. Participants will be informed that their participation is voluntary and that they may withdraw consent to participate at any time. Participants will be told that their records may be accessed by competent authorities and by authorized persons without violating the confidentiality of the participant, to the extent permitted by the applicable law(s) and/or regulations. By signing the Informed Consent Form (ICF), the participant is authorizing such access.

After this explanation and before entry to the study, written, dated, and signed informed consent will be obtained from the participant. The ICF will be provided in a language sufficiently understood by the participant. Participants are given the opportunity to ask questions. The participant will be given sufficient time to read the ICF and to ask additional questions. After this explanation and before entry to the study, consent will be appropriately recorded by means of the participant's dated signature. After having obtained the consent, a copy of the ICF is given to the participant.

15.3 Patient and Study Data Protection

An electronic case report form (eCRF) will be available in Qualtrics software. The eCRF will be completed for all participating patients. This eCRF will include specific pages for inclusion and exclusion

criteria, and for reporting each visit. Other specific pages will be dedicated to concomitant treatments and AEs (non-serious and serious). The investigator will review, approve, and validate each completed eCRF; the investigator's signature (validation) serving as attestation of the investigator's responsibility for ensuring that all data entered on the eCRF are complete, accurate and authentic.

All data will be processed according to the principles that the new European General Data Protection Regulation (GDPR) imposes, which are in force since 25 May 2018. The STIMULUS Research Group, Vrije Universiteit Brussel, will be responsible for the processing of personal data. The Data Protection officer is dpo@vub.be. The processing of personal data is for scientific research purposes and will happen on the legal basis of consent that can be withdrawn by the patient. All researchers involved in this clinical trial or in research projects that use materials original from this clinical trial are potential recipients of the personal data, as well as staff involved in monitoring and ethical evaluation and people from competent authorities and subcontracted parties that perform analysis on study-related data or materials. It is possible that personal data will be viewed by people who are in countries that do not use the same standards as the EU in terms of legal protection of data. In that case, we guarantee that the conditions of European and Belgian legislation on the protection of personal data will be respected. The study-related documents will be stored for at least 25 years.

15.4 Patient Identification

Patients who are included in the study will be assigned a unique study number. On all documents submitted to the sponsor, patients will only be identified by their study number. The patient identification list will be safeguarded. The name and any other directly identifying details will not be included in the study database.

16 Finance and Insurance

All patients will receive a financial compensation of 100 euro for study participation. Costs related to the hospital stay for tapering will be covered by the sponsor.

In accordance with the Belgian law relating to experiments in humans dated May 7, 2004, and/or with the Belgian law relating to clinical trials dated May 7, 2017 (as applicable) the Sponsor shall be liable, even without fault for any damages incurred by the Study Participant and linked directly or indirectly to the participation to the Study and the Sponsor shall provide compensation therefore through its insurance program. Before commencing the Study, the Sponsor shall enter an insurance contract which covers this liability, and the liability of every individual intervening in the Study, irrespective of the nature of the affiliation between the intervening individual, the Sponsor and the Study Participant. Every contractual provision aiming at limiting this liability is considered null and void.

The Participating Site, the Investigator and Sponsor shall have and maintain in full force and effect during the term of this Agreement (and following termination or completion of the Study to cover any claims arising from the Study) adequate insurance coverage for: (i) medical professional and/or medical malpractice liability, and (ii) general liability resulting from the Study at the Participating Site required by local law, each such insurance coverage in amounts appropriate to the conduct of the services of the Participating Site, the Investigator and Sponsor under this Agreement. The Participating Site and Sponsor shall be solely responsible for any deductible or self-insured retention under any such policies.

17 Reporting and Dissemination

If pain medication tapering programs are deemed to be more effective than no tapering, this would add to the evidence towards an improved patient-centered care model in this patient group and set

a clear path to advocate for pain medication tapering as the new standard treatment guideline for these patients before SCS. These scientific findings will be published in peer-reviewed scientific journals and can be directly translated into practice.

17.1 Steering and Advisory Board

The Steering Board is the main decision-making and steering body of the project. The Steering Board consists of the Principal Investigator (PI) Prof. Dr. Maarten Moens, Prof. Dr. Koen Putman, Prof. Dr. Cleo Crunelle, and Prof. Dr. Lisa Goudman. The Steering Board will organize a kick-off meeting at the start of the project to establish common working procedures. Furthermore, the Steering Board will hold meetings every three months. Additional teleconferences can be organized ad hoc in case of urgent issues. The main tasks of the Steering Board are: 1) Agree on common working procedures and management policies; 2) Monitor overall progress and follow-up of deliverables; 3) Milestone decisions; 4) Decisions on major changes to the work program; 5) Conflict handling; and 6) Budget decisions. The Steering Board is responsible for assuring the quality of the workflow and project implementation, taking into account the available resources. One of the most important tasks of the Steering Board is also to (re)direct all patients in the implementation of the opioid tapering program and the timely achievement of all deliverables.

The Advisory Committee (or valorization board) consists of the stakeholders who agreed to take part in the advisory committee. The advisory committee will support the dissemination & valorization leader in achieving the valorization goals and aid in organizing the various meetings foreseen for dissemination & valorization goals.

18 Conflict of Interest Statement

Funding is obtained by FWO – Fonds voor Wetenschappelijk Onderzoek – Vlaanderen, project code T000222N. STIMULUS research group received independent research grants from Medtronic. No conflicts of interest are present in the sponsor and other co-investigators. The authors and investigators declare that they have no competing interests.

19 Tables and Figures

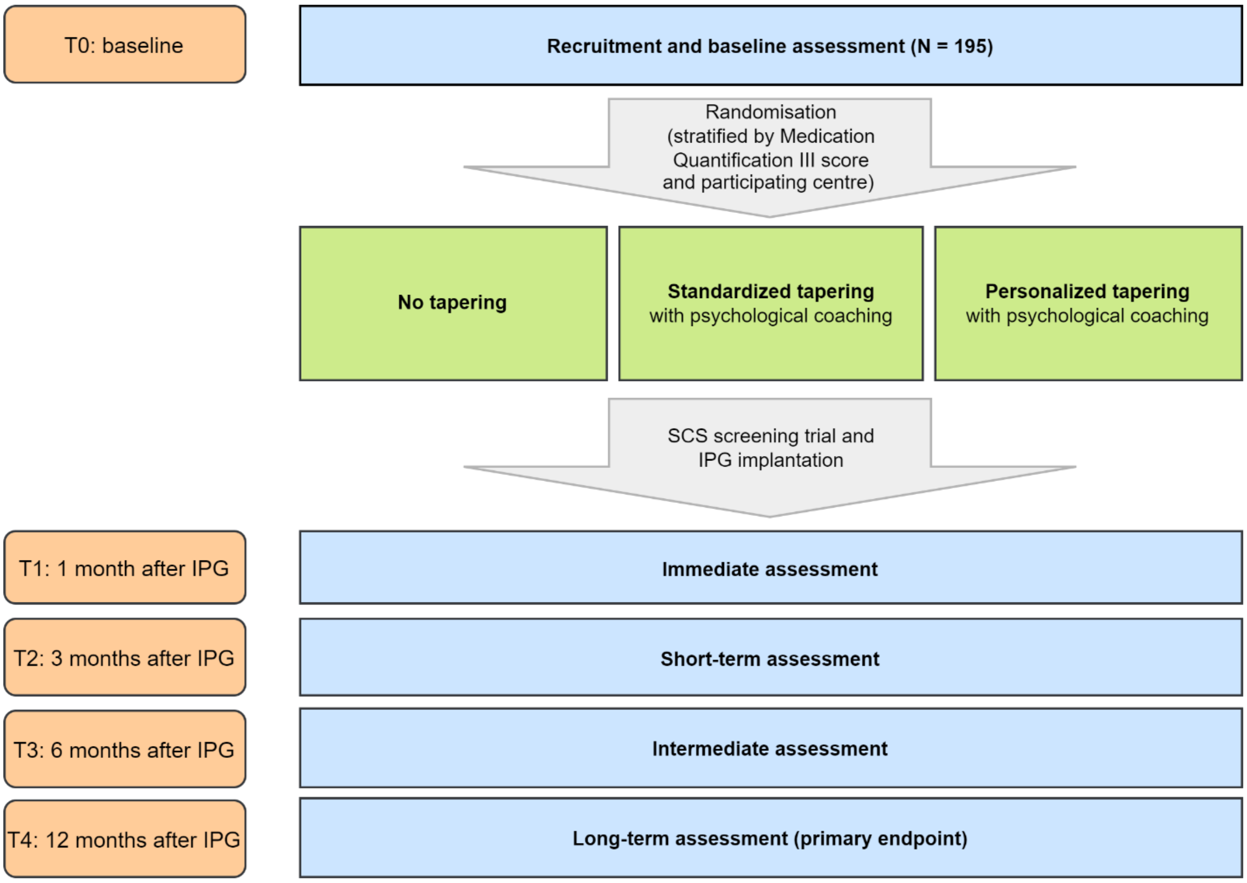

Figure 1: Study flowchart; Abbreviations: T = time point; SCS = Spinal Cord Stimulator; IPG = Implantable Pulse Generator

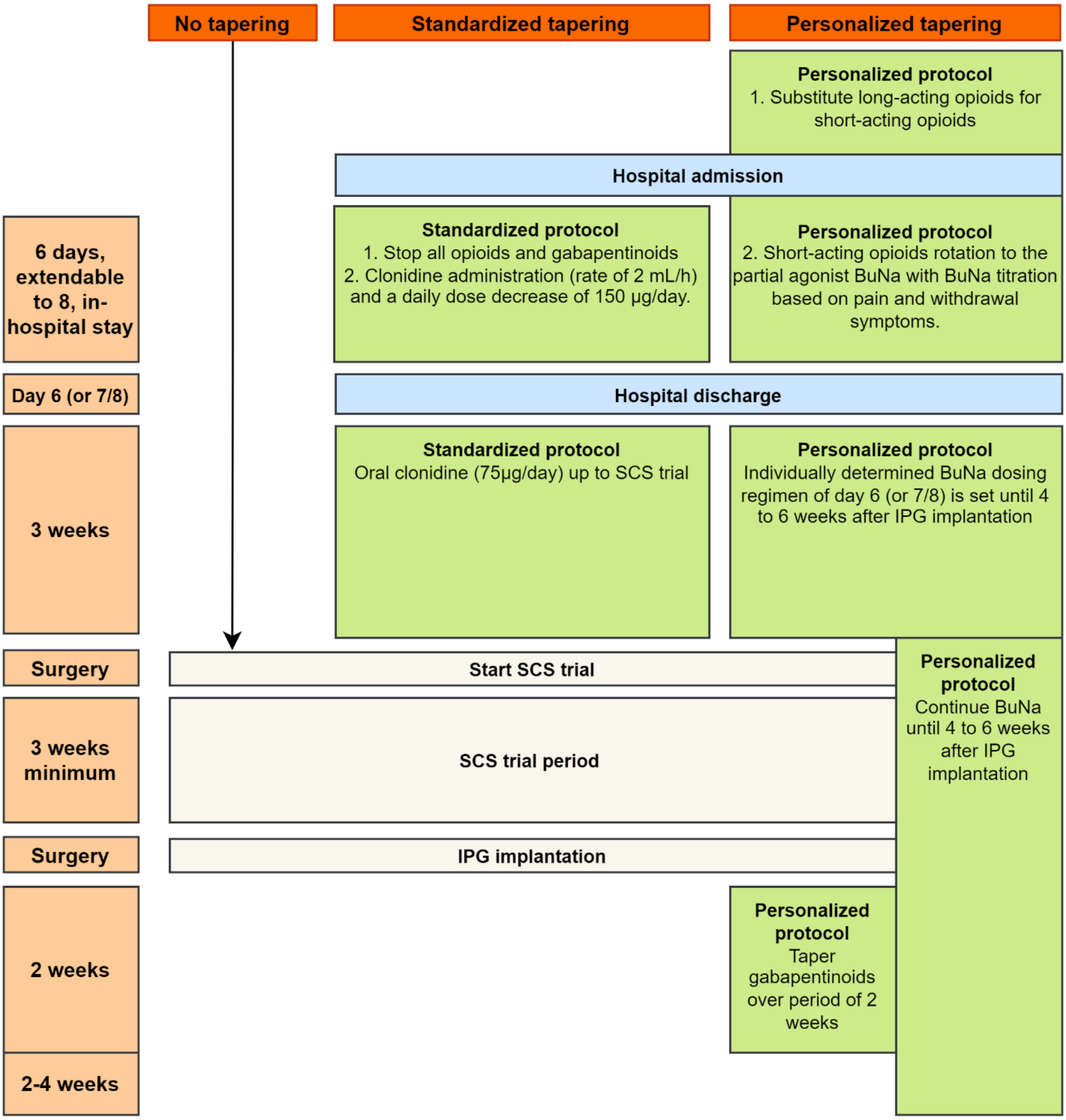

Figure 2: Overview of the three arms of this randomized controlled trial; Abbreviations: SCS = Spinal Cord Stimulator; IPG = Implantable Pulse Generator

<table>
<tr><th colspan="4"><u>During the hospital stay for tapering</u></th></tr>
<tr><td>D1</td><td>Stop all opioids and dilute 1200µg clonidine with saline (total of 48ml)
Start IV drip: 2ml/h
If needed: auxiliary medication in terms of symptom control</td><td rowspan="3">Check hemodynamic parameters</td><td rowspan="3">Psychological coaching</td></tr>
<tr><td>D2-6</td><td>Decrease daily clonidine dose by 150µg per day (or by 300µg if needed)
If needed: auxiliary medication in terms of symptom control
Pause drip if symptomatic hypotension or HR < 40 bpm
Restart drip at 1ml/h until normal hemodynamic parameters
Fluid challenge if hypotension without bradycardia
On D6: hospital discharge or extend hospital stay</td></tr>
<tr><td>D7-8</td><td>Decrease daily clonidine dose by 150µg per day (or by 300µg if needed)
If needed: auxiliary medication in terms of symptom control
Pause drip if symptomatic hypotension or HR < 40 bpm
Restart drip at 1ml/h until normal hemodynamic parameters
Fluid challenge if hypotension without bradycardia
On D7/8: hospital discharge</td></tr>
<tr><th colspan="4"><u>After the hospital stay for tapering</u></th></tr>
<tr><td>From hospital discharge to SCS-trial</td><td colspan="3">PO clonidine: 75µg per day</td></tr>
</table>

Figure 3: Schematic overview of standardized tapering; Abbreviations: D = day; IV = intravenous; HR = heart rate; PO = per os

Before the hospital stay for tapering		
Initiate switching long-acting opioid medication to dose-equivalent short-acting oxycodone		
During the hospital stay for tapering		
D1-2	Switching to dose-equivalent short-acting oxycodone	Psychological coaching
D3	Morning: Stop all opioid medication<br>Monitor withdrawal symptoms at least 4 times per day using COWS and SOS<br>Withdrawal symptoms (according to COWS/SOS): start 4/1mg BuNa<br>Re-evaluate withdrawal and if necessary: +2/0.5mg BuNa<br>Maximum daily dose: 24/6mg<br>If needed: auxiliary medication in terms of symptom control	
D4-5	Morning: give entire dose of previous day (if necessary: spread over max. 3x per day)<br>Monitor withdrawal symptoms at least 4 times per day using COWS and SOS<br>Re-evaluate withdrawal and if necessary: +2/0.5mg BuNa<br>Maximum daily dose: 36/9mg<br>If needed: auxiliary medication in terms of symptom control	
D6	Morning: give entire D5 dose (if necessary: spread over max. 3x per day)<br>Monitor withdrawal symptoms at least 4 times per day using COWS and SOS<br>Re-evaluate withdrawal and if necessary: +2/0.5mg BuNa<br>Maximum daily dose: 36/9mg<br>If needed: auxiliary medication in terms of symptom control<br>On D6: stabilization of BuNa and hospital discharge or extend hospital stay	
D7-8	Morning: give entire dose of previous day (if necessary: spread over max. 3x per day)<br>Monitor withdrawal symptoms at least 4 times per day using COWS and SOS<br>Re-evaluate withdrawal and if necessary: +2/0.5mg BuNa<br>Maximum daily dose: 36/9mg<br>If needed: auxiliary medication in terms of symptom control<br>On D7/8: stabilization of BuNa and hospital discharge	
After the hospital stay for tapering		
From hospital discharge to 4-6 weeks after definitive SCS implantation	Fixed dose BuNa (individually titrated in-hospital)	
4-6 weeks after definitive SCS implantation	BuNa tapering following underlying scheme	

	**BuNa dose (mg)**			
Day of BuNa tapering	**8/2mg**	**16/4mg**	**24/6mg**	**36/9mg**
1	6	12	20	24
2	6	12	20	24
3	6	10	16	20
4	4	10	16	20
5	4	10	12	16
6	4	8	12	16
7	2	8	12	12
8	2	8	8	12
9	2	4	8	10
10	2	4	8	10
11		4	4	8
12		2	4	8
13		2	4	6
14		2	2	6
15			2	4
16			2	4
17				4
18				2
19				2
20				2

Figure 4: Schematic overview of personalized tapering protocol; Abbreviations: D = day; COWS = Clinical Opiate Withdrawal Scale; SOS = Subjective Opioid Scale; BuNa = Buprenorphine/Naloxone

20 References

1. Sebaaly A, Lahoud M-J, Rizkallah M, Kreichati G, Kharrat K. Etiology, evaluation, and treatment of failed back surgery syndrome. *Asian spine journal*. 2018;12(3):574.
2. Chan CW, Peng P. Failed back surgery syndrome. *Pain Med*. Apr 2011;12(4):577-606. doi:10.1111/j.1526-4637.2011.01089.x
3. Hussain A, Erdek M. Interventional pain management for failed back surgery syndrome. *Pain Practice*. 2014;14(1):64-78.
4. Thomson S. Failed back surgery syndrome – definition, epidemiology and demographics. *British Journal of Pain*. 2013-02-01 2013;7(1):56-59. doi:10.1177/2049463713479096
5. Taylor RS, Taylor RJ. The economic impact of failed back surgery syndrome. *British journal of pain*. 2012;6(4):174-181.
6. Herman PM, Broten N, Lavelle TA, Sorbero ME, Coulter ID. Healthcare costs and opioid use associated with high-impact chronic spinal pain in the United States. *Spine*. 2019;44(16):1154.
7. Daniell JR, Osti OL. Failed back surgery syndrome: a review article. *Asian spine journal*. 2018;12(2):372.
8. Kalkman GA, Kramers C, van Dongen RT, van den Brink W, Schellekens A. Trends in use and misuse of opioids in the Netherlands: a retrospective, multi-source database study. *The Lancet Public Health*. 2019;4(10):e498-e505.
9. Dowell D, Haegerich TM, Chou R. CDC Guideline for Prescribing Opioids for Chronic Pain—United States, 2016. *JAMA*. 2016-04-19 2016;315(15):1624. doi:10.1001/jama.2016.1464
10. Dowell D, Haegerich TM. Using the CDC guideline and tools for opioid prescribing in patients with chronic pain. *American family physician*. 2016;93(12):970.
11. Philpot LM, Ramar P, Elrashidi MY, Mwangi R, North F, Ebbert JO. Controlled Substance Agreements for Opioids in a Primary Care Practice. *Journal of Pharmaceutical Policy and Practice*. 2017-12-01 2017;10(1)doi:10.1186/s40545-017-0119-5
12. D'Souza RS, Strand N. Neuromodulation With Burst and Tonic Stimulation Decreases Opioid Consumption: A Post Hoc Analysis of the Success Using Neuromodulation With BURST (SUNBURST) Randomized Controlled Trial. *Neuromodulation: Technology at the Neural Interface*. 2021/01/01/ 2021;24(1):135-141.
13. Al-Kaisy A, Van Buyten J-P, Carganillo R, et al. 10 kHz SCS therapy for chronic pain, effects on opioid usage: post hoc analysis of data from two prospective studies. *Scientific Reports*. 2019;9(1):1-11.
14. OECD. *Addressing Problematic Opioid Use in OECD Countries*. 2019.
15. Dunn KE, Weerts EM, Huhn AS, et al. Preliminary evidence of different and clinically meaningful opioid withdrawal phenotypes. *Addiction biology*. 2020;25(1):e12680.
16. Hutcheson D, Everitt B, Robbins T, Dickinson A. The role of withdrawal in heroin addiction: enhances reward or promotes avoidance? *Nature neuroscience*. 2001;4(9):943-947.
17. Chou R Fau - Turner JA, Turner Ja Fau - Devine EB, Devine Eb Fau - Hansen RN, et al. The effectiveness and risks of long-term opioid therapy for chronic pain: a systematic review for a National Institutes of Health Pathways to Prevention Workshop. (1539-3704 (Electronic))
18. Goudman L, De Smedt A, Forget P, Eldabe S, Moens M. High-dose spinal cord stimulation reduces long-term pain medication use in patients with failed back surgery syndrome who obtained at least 50% pain intensity and medication reduction during a trial period: a registry-based cohort study. *Neuromodulation: Technology at the Neural Interface*. 2021;24(3):520-531.
19. Waszak PM, Modrić M, Paturej A, et al. Spinal cord stimulation in failed back surgery syndrome: review of clinical use, quality of life and cost-effectiveness. *Asian spine journal*. 2016;10(6):1195.
20. Shealy CN, Mortimer JT, Reswick JB. Electrical inhibition of pain by stimulation of the dorsal columns: preliminary clinical report. *Anesthesia & Analgesia*. 1967;46(4):489-491.
21. Guan Y. Spinal cord stimulation: neurophysiological and neurochemical mechanisms of action. *Current pain and headache reports*. 2012;16(3):217-225.
22. Rigoard P, Basu S, Desai M, et al. Multicolumn spinal cord stimulation for predominant back pain in failed back surgery syndrome patients: a multicenter randomized controlled trial. *Pain*. 2019;160(6):1410.

23. Spinal cord stimulation for chronic pain of neuropathic or ischaemic origin. National Institute for Health and Care Excellence. Accessed 14/11/2022, 2022.
24. Gupta M, Abd-Elsayed A, Knezevic NN. Improving care of chronic pain patients with spinal cord stimulator therapy amidst the opioid epidemic. *Neurological Sciences*. 2020;41(10):2703-2710.
25. Remacle T, Mauviel S, Renwart HJ, et al. Long-Term Multicolumn-Lead Spinal Cord Stimulation Efficacy in Patients with Failed Back Surgery Syndrome: A Six-Year Prospective Follow-up Study. *World Neurosurg*. Oct 2020;142:e245-e252. doi:10.1016/j.wneu.2020.06.181
26. Niyomsri S, Duarte RV, Eldabe S, et al. A Systematic Review of Economic Evaluations Reporting the Cost-Effectiveness of Spinal Cord Stimulation. *Value in Health*. 2020-05-01 2020;23(5):656-665. doi:10.1016/j.jval.2020.02.005
27. Pollard EM, Lamer TJ, Moeschler SM, et al. <p>The effect of spinal cord stimulation on pain medication reduction in intractable spine and limb pain: a systematic review of randomized controlled trials and meta-analysis</p>. *Journal of Pain Research*. 2019-04-01 2019;Volume 12:1311-1324. doi:10.2147/jpr.s186662
28. Grider JS, Harned ME, Etscheidt MA. Patient selection and outcomes using a low-dose intrathecal opioid trialing method for chronic nonmalignant pain. *Pain physician*. Jul-Aug 2011;14(4):343-51.
29. Wilkes DM, Orillosa SJ, Hustak EC, et al. Efficacy, Safety, and Feasibility of the Morphine Microdose Method in Community-Based Clinics. *Pain Medicine*. 2018-09-01 2018;19(9):1782-1789. doi:10.1093/pm/pnx132
30. Gee L, Smith HC, Ghulam-Jelani Z, et al. Spinal Cord Stimulation for the Treatment of Chronic Pain Reduces Opioid Use and Results in Superior Clinical Outcomes When Used Without Opioids. *Neurosurgery*. Jan 1 2019;84(1):217-226. doi:10.1093/neuros/nyy065
31. Hov KR, Neerland BE, Andersen AM, et al. The use of clonidine in elderly patients with delirium; pharmacokinetics and hemodynamic responses. *BMC Pharmacology and Toxicology*. 2018-12-01 2018;19(1)doi:10.1186/s40360-018-0218-1
32. Hov KR, Neerland BE, Undseth Ø, et al. The Oslo Study of Clonidine in Elderly Patients with Delirium; LUCID: a randomised placebo-controlled trial. *International Journal of Geriatric Psychiatry*. 2019-07-01 2019;34(7):974-981. doi:10.1002/gps.5098
33. Jerjir A, Goudman L, Van Buyten JP, et al. Detoxification of Neuromodulation-Eligible Patients by a Standardized Protocol: A Retrospective Pilot Study. (1525-1403 (Electronic))
34. Veldman S, van Beek M, van Rijswijk S, et al. Effects of opioid rotation to buprenorphine/naloxone on pain, pain thresholds, pain tolerance, and quality of life in patients with chronic pain and opioid use disorder. *Pain*. 2022/05// 2022;163(5):955-963. doi:10.1097/j.pain.0000000000002462
35. Schellekens AFA, Veldman SE, Suranto ESD, et al. Beneficial Effects of Opioid Rotation to Buprenorphine/Naloxone on Opioid Misuse, Craving, Mental Health, and Pain Control in Chronic Non-Cancer Pain Patients with Opioid Use Disorder. *Journal of Clinical Medicine*. 2021-08-21 2021;10(16):3727. doi:10.3390/jcm10163727
36. Ling W, Hillhouse M, Domier C, et al. Buprenorphine tapering schedule and illicit opioid use. *Addiction*. 2009/02// 2009;104(2):256-265. doi:10.1111/j.1360-0443.2008.02455.x
37. Schulman JJ, Ramirez RR, Zonenshayn M, Ribary U, Llinas R. Thalamocortical dysrhythmia syndrome: MEG imaging of neuropathic pain. *Thalamus & Related Systems*. 2005;3(1):33-39. doi:10.1017/S1472928805000063
38. Sheehan DV, Lecrubier Y, Sheehan KH, et al. The Mini-International Neuropsychiatric Interview (M.I.N.I.): the development and validation of a structured diagnostic psychiatric interview for DSM-IV and ICD-10. *J Clin Psychiatry*. 1998;59 Suppl 20:22-33;quiz 34-57.
39. Chapman JR, Norvell DC, Hermsmeyer JT, et al. Evaluating Common Outcomes for Measuring Treatment Success for Chronic Low Back Pain. *Spine*. 2011;36:S54-S68. doi:10.1097/BRS.0b013e31822ef74d
40. Fairbank JCT, Pynsent PB. The Oswestry Disability Index. *Spine*. 2000/11/15/ 2000;25(22):2940-2953.
41. Park KB, Shin J-S, Lee J, et al. Minimum Clinically Important Difference and Substantial Clinical Benefit in Pain, Functional, and Quality of Life Scales in Failed Back Surgery Syndrome Patients. *Spine*. 2017;42(8):E474-E481. doi:10.1097/brs.0000000000001950

42. Jensen MP, Karoly P, Braver S. The measurement of clinical pain intensity: a comparison of six methods. *Pain*. 1986/10/01/ 1986;27(1):117-126.
43. Ogon M, Krismer M, Söllner W, Kantner-Rumplmair W, Lampe A. Chronic low back pain measurement with visual analogue scales in different settings. *Pain*. 1996/03/01/ 1996;64(3):425-428.
44. Hägg O, Fritzell P, Nordwall A. The clinical importance of changes in outcome scores after treatment for chronic low back pain. *European Spine Journal*. 2003-02-01 2003;12(1):12-20. doi:10.1007/s00586-002-0464-0
45. Ferraz MB, Quaresma MR, Aquino LR, Atra E, Tugwell P, Goldsmith CH. Reliability of pain scales in the assessment of literate and illiterate patients with rheumatoid arthritis. *The Journal of rheumatology*. 1990/08// 1990;17(8):1022-1024.
46. Herdman M, Gudex C, Lloyd A, et al. Development and preliminary testing of the new five-level version of EQ-5D (EQ-5D-5L). *Quality of Life Research*. 2011-12-01 2011;20(10):1727-1736. doi:10.1007/s11136-011-9903-x
47. Cheung PWH, Wong CKH, Cheung JPY. Differential Psychometric Properties of EuroQoL 5-Dimension 5-Level and Short-Form 6-Dimension Utility Measures in Low Back Pain. *Spine*. 2019;44(11):E679-E686. doi:10.1097/brs.0000000000002939
48. Janssen MF, Pickard AS, Golicki D, et al. Measurement properties of the EQ-5D-5L compared to the EQ-5D-3L across eight patient groups: a multi-country study. *Quality of Life Research*. 2013-09-01 2013;22(7):1717-1727. doi:10.1007/s11136-012-0322-4
49. Bouckaert N, Cleemput I, Devriese S, Gerkens S. An EQ-5D-5L Value Set for Belgium. *PharmacoEconomics - Open*. 2022-11-01 2022;6(6):823-836. doi:10.1007/s41669-022-00353-3
50. Cardol M, de Haan RJ, van den Bos GAM, de Jong BA, de Groot IJM. The development of a handicap assessment questionnaire: the Impact on Participation and Autonomy (IPA). *Clinical Rehabilitation*. 1999;13(5):411-419. doi:10.1191/026921599668601325
51. Cardol M, Beelen A, Van Den Bos GA, De Jong BA, De Groot IJ, De Haan RJ. Responsiveness of the impact on participation and autonomy questionnaire. *Archives of Physical Medicine and Rehabilitation*. 2002-11-01 2002;83(11):1524-1529. doi:10.1053/apmr.2002.35099
52. Cardol M, de Haan RJ, de Jong BA, van den Bos GAM, de Groot IJM. Psychometric properties of the impact on Participation and Autonomy Questionnaire. *Archives of Physical Medicine and Rehabilitation*. 2001/02/01/ 2001;82(2):210-216.
53. Schippers GM, Broekman TG, Buchholz A, Koeter MWJ, Van Den Brink W. Measurements in the Addictions for Triage and Evaluation (MATE): an instrument based on the World Health Organization family of international classifications. *Addiction*. 2010-05-01 2010;105(5):862-871. doi:10.1111/j.1360-0443.2009.02889.x
54. Boyett B, Wiest K, McLeod LD, et al. Assessment of craving in opioid use disorder: Psychometric evaluation and predictive validity of the opioid craving VAS. *Drug Alcohol Depend*. Dec 1 2021;229(Pt B):109057. doi:10.1016/j.drugalcdep.2021.109057
55. Butler SF, Budman SH, Fanciullo GJ, Jamison RN. Cross Validation of the Current Opioid Misuse Measure to Monitor Chronic Pain Patients on Opioid Therapy. *The Clinical Journal of Pain*. 2010-11-01 2010;26(9):770-776. doi:10.1097/ajp.0b013e3181f195ba
56. Masters Steedman S, Middaugh Sj Fau - Kee WG, Kee Wg Fau - Carson DS, Carson Ds Fau - Harden RN, Harden Rn Fau - Miller MC, Miller MC. Chronic-pain medications: equivalence levels and method of quantifying usage. (0749-8047 (Print))
57. Gallizzi M, Gagnon C, Harden RN, Stanos S, Khan A. Medication Quantification Scale Version III: Internal Validation of Detriment Weights Using a Chronic Pain Population. *Pain Practice*. 2008-01-01 2008;8(1):1-4. doi:10.1111/j.1533-2500.2007.00163.x
58. Harden RN, Weinland SR, Remble TA, et al. Medication Quantification Scale Version III: Update in Medication Classes and Revised Detriment Weights by Survey of American Pain Society Physicians. *The Journal of Pain*. 2005/06/01/ 2005;6(6):364-371.
59. Zigmond AS, Snaith RP. The hospital anxiety and depression scale. *Acta Psychiatr Scand*. Jun 1983;67(6):361-70. doi:10.1111/j.1600-0447.1983.tb09716.x

60. Bjelland I, Dahl AA, Haug TT, Neckelmann D. The validity of the Hospital Anxiety and Depression Scale. An updated literature review. *J Psychosom Res*. Feb 2002;52(2):69-77. doi:10.1016/s0022-3999(01)00296-3
61. Hott A, Pripp AH, Juel NG, Liavaag S, Brox JI. Self-efficacy and Emotional Distress in a Cohort With Patellofemoral Pain. *Orthop J Sports Med*. Mar 2022;10(3):23259671221079672. doi:10.1177/23259671221079672
62. Luszczynska A, Scholz U, Schwarzer R. The general self-efficacy scale: multicultural validation studies. *J Psychol*. Sep 2005;139(5):439-57. doi:10.3200/jrlp.139.5.439-457
63. Samulowitz A, Hensing G, Haukenes I, Bergman S, Grimby-Ekman A. General self-efficacy and social support in men and women with pain - irregular sex patterns of cross-sectional and longitudinal associations in a general population sample. *BMC Musculoskelet Disord*. Nov 29 2022;23(1):1026. doi:10.1186/s12891-022-05992-5
64. Van der Maas L, De Vet H, Köke A, Bosscher R, Peters M. Psychometric Properties of the Pain Self-Efficacy Questionnaire (PSEQ) Validation, Prediction, and Discrimination Quality of the Dutch Version. *European Journal of Psychological Assessment*. 2012/09/01/ 2012;28:68-75. doi:10.1027/1015-5759/a000092
65. Lacasse A, Bourgault P, Tousignant-Laflamme Y, Courtemanche-Harel R, Choinière M. Development and validation of the French-Canadian Chronic Pain Self-efficacy Scale. *Pain Res Manag*. Mar-Apr 2015;20(2):75-83. doi:10.1155/2015/832875
66. Büssing A, Poier D, Ostermann T, Kröz M, Michalsen A. Treatment of Chronic Lower Back Pain: Study Protocol of a Comparative Effectiveness Study on Yoga, Eurythmy Therapy, and Physiotherapeutic Exercises. *Complement Med Res*. 2018;25(1):24-29. doi:10.1159/000471801
67. Rashid M, Kristofferzon ML, Heiden M, Nilsson A. Factors related to work ability and well-being among women on sick leave due to long-term pain in the neck/shoulders and/or back: a cross-sectional study. *BMC Public Health*. May 30 2018;18(1):672. doi:10.1186/s12889-018-5580-9
68. Sullivan MJ, Bishop SR, Pivik J. The pain catastrophizing scale: development and validation. *Psychological assessment*. 1995;7(4):524.
69. Van Damme S CG, Vlaeyen JWS, Goubert L, Van den Broeck A,, B VH. De Pain Catastrophizing Scale: psychometrische karakteristieken en normering. *Gedragstherapie*. 2000;3:211–222.
70. Ikemoto T, Hayashi K, Shiro Y, et al. A systematic review of cross-cultural validation of the pain catastrophizing scale. *Eur J Pain*. Aug 2020;24(7):1228-1241. doi:10.1002/ejp.1587
71. Paroli M, Bernini O, De Carolis G, et al. Are Multidimensional pain inventory coping strategy profiles associated with long-term spinal cord stimulation effectiveness? *Pain Medicine*. 2018;19(5):1023-1032.
72. Carpenter JS, Andrykowski MA. Psychometric evaluation of the Pittsburgh sleep quality index. *Journal of psychosomatic research*. 1998;45(1):5-13.
73. Buysse DJ, Reynolds III CF, Monk TH, Berman SR, Kupfer DJ. The Pittsburgh Sleep Quality Index: a new instrument for psychiatric practice and research. *Psychiatry research*. 1989;28(2):193-213.
74. Phelps C, Bellon S, Hinkey M, et al. Measurement properties of Patient-Reported Outcome Measures used to assess the sleep quality in adults with high prevalence chronic pain conditions: a systematic review. *Sleep Med*. Oct 2020;74:315-331. doi:10.1016/j.sleep.2020.06.028
75. Mayer TG, Neblett R, Cohen H, et al. The Development and Psychometric Validation of the Central Sensitization Inventory. *Pain Practice*. 2012-04-01 2012;12(4):276-285. doi:10.1111/j.1533-2500.2011.00493.x
76. Neblett R, Cohen H, Choi Y, et al. The Central Sensitization Inventory (CSI): establishing clinically significant values for identifying central sensitivity syndromes in an outpatient chronic pain sample. *The journal of pain : official journal of the American Pain Society*. May 2013;14(5):438-45. doi:10.1016/j.jpain.2012.11.012
77. Goudman L, De Smedt A, Eldabe S, et al. High-dose spinal cord stimulation for patients with failed back surgery syndrome: a multicenter effectiveness and prediction study. *PAIN*. 2021;162(2):582-590. doi:10.1097/j.pain.0000000000002035
78. Chow S-C, Shao J, Wang H, Lokhnygina Y. *Sample size calculations in clinical research*. vol Third edition. Chapman & Hall/CRC biostatistics series. Taylor & Francis Group; 2018.
79. Ellerbroek H, Van Den Heuvel SAS, Dahan A, Timmerman H, Kramers C, Schellekens AFA. Buprenorphine/naloxone versus methadone opioid rotation in patients with prescription opioid use disorder

and chronic pain: study protocol for a randomized controlled trial. *Addiction Science & Clinical Practice*. 2022-09-04 2022;17(1)doi:10.1186/s13722-022-00326-1
80. de Jong C, van Hoek A, Jongerhuis M, Fiers M, Ghijsen L, Gottmer P. Richtlijn detox. 2004.
81. Barber JP, Liese, B.S., Abrams, M.,J. Development of the Cognitive Therapy Adherence and Competence Scale. *Psychotherapy Research*. 2003;13:205-221.
82. Reme SE, Tveito TH, Harris A, et al. Cognitive Interventions and Nutritional Supplements (The CINS Trial): A Randomized Controlled, Multicenter Trial Comparing a Brief Intervention With Additional Cognitive Behavioral Therapy, Seal Oil, and Soy Oil for Sick-Listed Low Back Pain Patients. *Spine*. Oct 15 2016;41(20):1557-1564. doi:10.1097/brs.0000000000001596
83. Mowbray C, Holter M, Teague G, Bybee D. Fidelity Criteria: Development, Measurement, and Validation. *The American Journal of Evaluation*. 2003;24(3):315-340. doi:10.1016/s1098-2140(03)00057-2
84. Ramsey SD, Willke RJ, Glick H, et al. Cost-effectiveness analysis alongside clinical trials II—an ISPOR Good Research Practices Task Force report. *Value in Health*. 2015;18(2):161-172.
85. Cleemput INM, Van de Sande S, Thiry N. Belgische richtlijnen voor economische evaluaties en budget impact analyses: tweede editie. Health technology assessment (HTA). Federaal Kenniscentrum voor de Gezondheidszorg (KCE) Brussel; 2012.
86. Glick H, Doshi J, Sonnad S, Polsky D. Economic Evaluation in Clinical Trials: Oxford University Press. *Kettering, UK*. 2014;
87. Husereau D, Drummond M, Augustovski F, et al. Consolidated Health Economic Evaluation Reporting Standards 2022 (CHEERS 2022) statement: updated reporting guidance for health economic evaluations. *International Journal of Technology Assessment in Health Care*. 2022;38(1)
88. Siebert U, Alagoz O, Bayoumi AM, et al. State-transition modeling: a report of the ISPOR-SMDM Modeling Good Research Practices Task Force-3. *Med Decis Making*. Sep-Oct 2012;32(5):690-700. doi:10.1177/0272989x12455463
89. French C, Pinnock H, Forbes G, Skene I, Taylor SJ. Process evaluation within pragmatic randomised controlled trials: what is it, why is it done, and can we find it?—a systematic review. *Trials*. 2020;21(1):1-16.
90. Skivington K, Matthews L, Simpson SA, et al. A new framework for developing and evaluating complex interventions: update of Medical Research Council guidance. *bmj*. 2021;374
91. Grant A, Treweek S, Dreischulte T, Foy R, Guthrie B. Process evaluations for cluster-randomised trials of complex interventions: a proposed framework for design and reporting. *Trials*. 2013;14(1):1-10.
